# Time-Resolved Electrochemical Surface-Enhanced Resonance Raman Spectroscopy Double-Fingerprint for the Detection of *Pseudomonas aeruginosa*

**DOI:** 10.1021/acsmeasuresciau.6c00144

**Published:** 2026-07-30

**Authors:** Luis Romay, Esther Carcelen, Sandra de la Parra, Sara Collado, Rocio Barros, Carlos Rumbo, Aranzazu Heras, Alvaro Colina

**Affiliations:** † Department of Chemistry, 16725 Universidad de Burgos, Plaza Misael Bañuelos s/n, E-09001 Burgos, Spain; ‡ International Research Center in Critical Raw Materials for Advanced Industrial Technologies-ICCRAM, Universidad de Burgos, Plaza Misael Bañuelos s/n, E-09001 Burgos, Spain

**Keywords:** EC-SERS, bacteria, Pseudomonas aeruginosa, pyocyanin, electrochemistry

## Abstract

Bacterial detection constitutes a significant area of contemporary research. A particularly compelling strategy for unequivocal bacterial identification involves measuring specific biomarkers. In the context of bacteria responsible for infections, the development of rapid and highly sensitive analytical detection methods is crucial for timely application of necessary medical treatments. Time-resolved electrochemical surface-enhanced Raman scattering (TR-EC-SERS) has emerged as a promising technique for addressing these challenges. This work presents a TR-EC-SERS-based methodology for the detection of pyocyanin, a biomarker of *Pseudomonas aeruginosa*. TR-EC-SERS allows the detection of pyocyanin at picomolar concentrations in synthetic samples and nanomolar concentrations in complex media. In fact, pyocyanin exhibits pronounced surface-enhanced resonance Raman scattering (SERRS) characteristics, which enhance the sensitivity of the analytical method. Moreover, time-resolved electrochemical surface-enhanced resonance Raman scattering (TR-EC-SERRS) provides a double fingerprint response based on the Raman spectrum and the shape of the corresponding voltaRamangram, which depends on the electrochemical response of pyocyanin, showing a very high selectivity for pyocyanin and demonstrating the advantages of this technique for obtaining reliable information in the study of complex chemical systems. Furthermore, TR-EC-SERRS facilitates the monitoring of pyocyanin secretion during the growth of *P. aeruginosa* in a culture medium, providing significant insights into this process. A simple pretreatment of bacterial samples based on the use of ether is proposed, which inactivates *P. aeruginosa* and facilitates its subsequent analysis.

## Graphical abstract

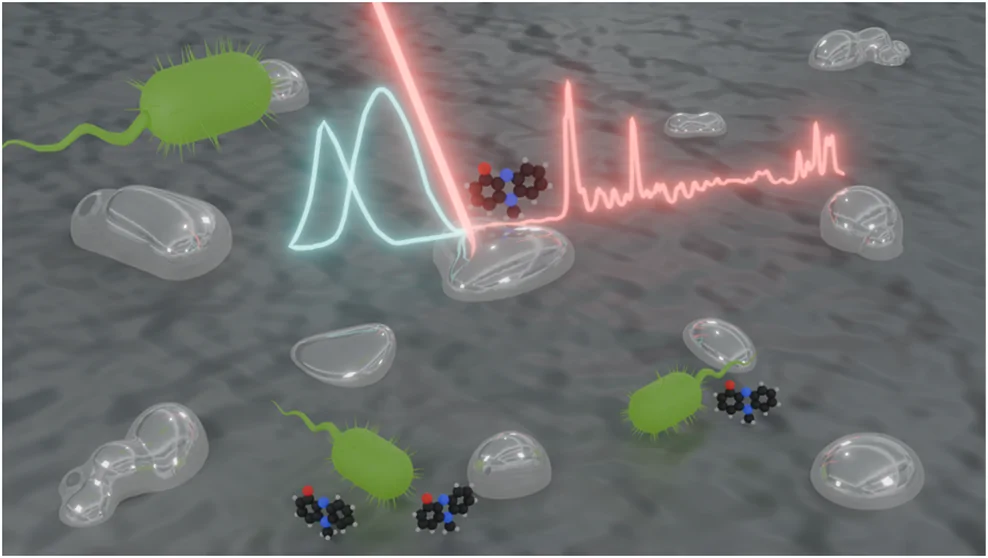

## Introduction

*Pseudomonas aeruginosa* (*PA*) is a Gram-negative bacterium that has been designated by the World Health Organization (WHO) as a priority antibiotic-resistant bacterial pathogen.^1,2^ This designation is indicative of its clinical relevance and its capacity to develop multidrug resistance, as well as its persistence in both hospital and community environments.^1–5^ Although *PA* rarely causes disease in healthy individuals, its incidence increases substantially in immunocompromised patients, individuals with open wounds or extensive burns, and patients with implanted medical devices.^6–8^ In this context, *PA* is regarded as an opportunistic pathogen and an important cause of nosocomial infections, with a major clinical impact across respiratory, urinary, vascular, and central nervous system infections.^9–12^

The success of *PA* as a pathogen is linked to a broad repertoire of virulence factors,^6^ including robust biofilm formation^9,12,13^ and coordinated regulation through *quorum-sensing* (QS) networks,^14^ which together promote persistence and immune evasion. This observation underscores the imperative for timely and reliable diagnostic methods.^15–18^ Among the *PA* virulence-associated metabolites, phenazines represent a family of pigmented, redox-active compounds. These compounds are produced, particularly during the early stages of biofilm development.^19,20^ Pyocyanin (PYO) is the most extensively studied phenazine within this family, and interest in this molecule has increased in recent years due to its biological relevance and potential applications.^21–24^ PYO has been identified as a specific biomarker of *PA*,^6,23–25^ with *PA* being the only known organism capable of naturally producing PYO.^25^

Building on this premise, various methodologies have been developed to detect the presence of *PA*, often by targeting PYO as a diagnostic marker. Traditionally, PYO analysis in clinical samples has involved the chloroform extraction of the pigment, followed by quantification using different analytical techniques, such as high-performance liquid chromatography (HPLC)^26–28^ or alternative methods.^29,30^ The growing interest in PYO over the past decade has further driven the development of increasingly selective and sensitive detection strategies, including chromatographic approaches,^31^ optical techniques^32,33^ and particularly electrochemical methods.^34–39^ However, many of these approaches remain limited by long analysis times and high costs, which are frequently linked to what is widely considered the main bottleneck in bioanalysis: sample preparation.^40,41^ This is particularly critical because each biological matrix presents unique challenges and sources of interference in the analysis. Recent advances in electrochemical methods are driving a clear trend toward point-of-care diagnostics by eliminating the need for complex sample pretreatment and enabling rapid analysis within minutes with very competitive limits of detection in the order of nanomolar. Portable devices simplify the detection through direct contact with the sample or diffusion across a hydrogel layer. The main limitation of the electrochemical methods is related to the lack of molecular specificity which can be obtained with optical or mass spectroscopy. Conventional techniques such as HPLC-MS/MS require labor-intensive solvent extraction and column-cleaning procedures before analysis. In the case of the colorimetric detection, both the limits of detection and the analysis time are less competitive than other analytical techniques. The development of simple pretreatments combined with sensitive and selective analytical techniques, such as SERS, which provides specific molecular information, facilitates the detection of molecules in complex media.

Operando techniques, such as time-resolved electrochemical surface-enhanced Raman scattering (TR-EC-SERS), are powerful tools for obtaining complementary molecular information during electrochemical and bioanalytical processes under realistic working conditions.^42–44^ Furthermore, the Raman intensity is increased by several orders of magnitude when the laser wavelength is in resonance with an electronic transition of the analyte, giving rise to surface-enhanced resonance Raman scattering (SERRS).^45–47^ In this context, TR-EC-SERS has emerged as a straightforward approach to address several limitations of conventional methodologies by combining the strengths of electrochemistry (EC) with the molecular specificity and sensitivity of SERS.^42^ TR-EC-SERS can amplify the Raman signal of molecules located in the vicinity of plasmonic nanostructures, yielding highly sensitive vibrational “fingerprints” that enable qualitative identification and, in many cases, quantitative analysis, even in complex matrices.^42^ In recent years, advances in electrochemically generated SERS-active substrates, together with the development and adoption of screen-printed electrodes (SPEs), have further improved the reproducibility and sensitivity of EC-SERS-based methods.^44,48–54^

The growing interest in PYO has driven the development of new analytical strategies for its determination, including methodologies based on SERS and EC-SERS.^30,32,47,55–58^ Bohn et al. investigated the behavior of PYO using EC-SERS, studying the influence of pH on the Raman/SERS fingerprint, the electrochemical response at different pH values, and the spectroelectrochemical behavior under applied potential,^58^ obtaining a very good characterization of the *PA*. Nevertheless, reliable quantification in biological samples remains challenging, largely due to the demanding sample pretreatments and, in many cases, the need for prior nanoparticle synthesis. In this work, we addressed these limitations using complementary approaches. A simple sample-handling protocol is proposed: samples that may contain *PA* are treated with ether 1:1 (v/v), after which the solvent is allowed to evaporate before SERS measurements. This pretreatment inactivates *PA*, enabling safer handling of biological samples in settings where direct manipulation of viable bacteria would not normally be appropriate, while avoiding the potential interference of ether with the measurement workflow. A time-resolved operando strategy for PYO detection is proposed, which not only enables the *in situ* formation of SERS-active nanoparticles in a simple and reproducible way, but also, taking into account that PYO is a redox-active molecule that can be electrochemically reduced,^34–39^ allows the acquisition of a potential dependent SERS response, which is characteristic of the target molecule. In time-resolved Raman spectroelectrochemical measurements, redox conversions can be translated into measurable and dynamic changes in the SERS intensity. This is particularly valuable for highly complex samples because it provides not only the characteristic vibrational spectrum of the target molecule but also a potential-dependent response that shows how the SERS signal evolves according to the applied potential. Together, these two pieces of information provide a “double fingerprint” that strengthens the molecular detection. Using this workflow, we have achieved competitive limits of detection (LOD) in aqueous samples and have demonstrated that the method remains effective for PYO detection across diverse matrices, including direct bacterial cultures, tap water, saliva, urine, and serum, yielding competitive results compared to the analytical results reported in the literature.

## Materials and Methods

### Chemicals and Materials

Acetic acid (C_2_H_4_O_2_, 100%, reagent, VWR), adenine (C_5_H_5_N_5_, 99+ %, reagent, Sigma-Aldrich), boric acid (H_3_BO_3_, 99%, reagent, Panreac), dimethyl ether (C_2_H_6_O, 99.8+ %, reagent, VWR), folic acid (C_19_H_19_N_7_O_6_, 97%, reagent, Sigma-Aldrich), lithium perchlorate (LiClO_4_, 99+ %, reagent, ACROS Organics), Mueller-Hinton Broth (OXOID), Mueller-Hinton Agar (Scharlau), orthophosphoric acid (H_3_PO_3_, 85%, reagent, Panreac), potassium chloride (KCl, 99+ %, reagent, ACROS Organics), pyocyanin (PYO, C_13_H_10_N_2_O, 98+ % reagent, Sigma-Aldrich), sodium hydroxide (NaOH, 99+ %, reagent, ACROS Organics) were analytical grade and used as received without further purification. Human serum was obtained from Sigma-Aldrich (Human Serum Heat Inactivated, from human male AB plasma, sterile-filtered, H3667–20 ML, SLCQ8302), Surine E (710) was obtained from DYNA-TEK, INC, and saliva was obtained from a 30-year-old male with a varied diet and free of other drugs. Aqueous solutions were freshly prepared using ultrapure water (18.2 MΩ cm resistivity at 25 °C, 2 ppb TOC, Milli-Q SQ 2Series water purification system, Merck-Millipore).

### Bacterial Strain and Culture Conditions

*P. aeruginosa* PAO1 was used in this study. Unless otherwise stated, the following culture protocol was used to obtain cultures with a controlled optical density (OD_600_) for subsequent measurements, as shown in Figure S1. A glycerol stock of *PA* (initial sample) was streaked onto Mueller–Hinton (MH) agar plates (first plating) and incubated for 24 h at 37 °C. A single colony from the first plate was restreaked onto a fresh MH agar plate (second plating) and incubated for 24 h at 37 °C. After the second plating, a single colony was transferred to MH broth in a sterile conical tube and incubated for 24 h at 37 °C with shaking at 180 rpm (preinoculum). The OD_600_ of the preinoculum was then measured, and the culture was diluted to an OD_600_ of 0.04 in fresh MH broth. This diluted culture was subsequently incubated in MH broth at 37 °C and 180 rpm, and aliquots were withdrawn at the required time points for measurements.

### Sample Preparation

Unless otherwise stated, all biological samples were processed immediately using ether at a 1:1 (v/v) ratio. For samples obtained from liquid cultures, the culture was vigorously mixed, and a 0.5 mL aliquot was transferred into an Eppendorf tube. Subsequently, 0.5 mL of ether (1:1, v/v) was added, and the mixture was shaken vigorously. The ether phase was subsequently allowed to evaporate at room temperature for at least 30 min prior to analysis, and complete evaporation was visually confirmed by ensuring that only the aqueous phase remained for the analysis. Figure S2 summarizes the sample preparation process.

### Instrumentation

Operando Raman measurements were conducted using a customized SPELEC RAMAN (Metrohm-DropSens S.L.) with a 785 nm laser source operating at a power of 203 mW (642 W cm^–2^), unless otherwise stated. Time-resolved UV/vis absorption SEC (TR-UV/vis-SEC) experiments were performed using a SPELEC (Metrohm-DropSens S.L.) customized instrument. This device includes deuterium (215–400 nm) and tungsten-halogen (360–2500 nm) light sources. DropView SPELEC (Metrohm-DropSens S.L.) was the software used to acquire the spectroelectrochemical data. A different silver SPE (Ag-SPE, DRP-C013, Metrohm-DropSens S.L.) was used in each Raman spectroelectrochemical experiment. The Ag-SPEs consisted of three electrodes: a silver working electrode (WE), a pseudoreference electrode (RE), and a carbon counter electrode (CE). A different gold SPE (Au-SPE, 220AT, Metrohm-DropSens S.L.) was used for each TR-UV/vis-SEC experiment. The Au-SPEs consisted of three electrodes: a gold working electrode (WE), a gold counter electrode (CE), and a silver pseudoreference electrode (RE). Data treatment was performed using MATLAB R2024b.

### Spectroelectrochemistry (SEC) Measurements

Cyclic voltammetry (CV) and linear sweep voltammetry (LSV) were selected as the electrochemical techniques for the spectroelectrochemical (SEC) experiments. A pretreatment of the Ag-SPE, as described in Figure S3, was required for all the TR-EC-SERS measurements to obtain good reproducibility and Raman signal enhancement.^51^ All experiments started with a CV preconditioning step in 0.1 M LiClO_4_ and 10 mM KCl to modify the initial surface with AgCl seeds, facilitating the generation of SERS-active silver nanoparticles (Ag NPs) in the detection stage, compared to direct measurements performed without preconditioning. The CV vertex potentials were set to +0.20 V and −0.20 V. After a 5 s equilibration time at the starting potential, the potential scan began at +0.20 V and continued in the cathodic direction at a scan rate of 0.02 V·s^–1^ for two cycles (Figure S3). After preconditioning, the SPE was thoroughly rinsed with deionized water prior to the measurement step. For the measurement stage, experiments were carried out in a medium similar to that used during the preconditioning step (0.1 M LiClO_4_ and 10 mM KCl), but additionally containing Britton-Robinson Buffer (BRB, pH = 6) and different concentrations of PYO. The LSV was recorded after a 5 s equilibration time at the starting potential, scanning from +0.20 V to – 0.50 V in the cathodic direction at 0.02 V·s^–1^ (Figure S3A). In some cases, when applicable, a CV was used for this measurement under the same conditions as the LSV (Figure S3B). All potentials are reported with respect to the Ag pseudo-RE integrated into the SPE. Unless otherwise specified, the integration time for obtaining each Raman spectrum was 1 s.

### Scanning Electron Microscope Images

A JEOL JSM IT700HR field-emission scanning electron microscope (FE-SEM) was used to obtain scanning electron microscope images of the WE surface in different states. An electron beam of 3 kV was used with an in-lens secondary electron detector.

## Results and Discussion

### Spectroelectrochemistry Analysis of Pyocyanin

Before starting the analysis of PYO, it is important to understand the electrochemical behavior of this molecule. For this purpose, TR-EC-SERS and TR-UV/vis-SEC experiments were performed using a Ag-SPE and a Au-SPE, respectively. Figure 1 summarizes the SEC responses of these experiments obtained by CV performed between the vertex potentials of +0.20 and −0.50 V, starting at +0.20 V in the cathodic direction with a scan rate of 0.02 V·s^–1^ in a solution containing 18.5 nM PYO, BRB at pH = 6, 0.1 M LiClO_4_, and 10 mM KCl for the EC-SERS experiment. Similar conditions were used for the UV/vis absorption SEC experiment, except for the higher PYO concentration (555 μM) employed to obtain a good optical and electrochemical signal. At the nanomolar concentration range used for EC-SERS, the PYO response is not clearly resolved by direct CV or UV/vis absorption. The protocol used for EC-SERS is described in Figure S3, including a pretreatment step, which generates AgCl seeds on the electrode surface to facilitate the synthesis of Ag NPs.

**Figure 1:**
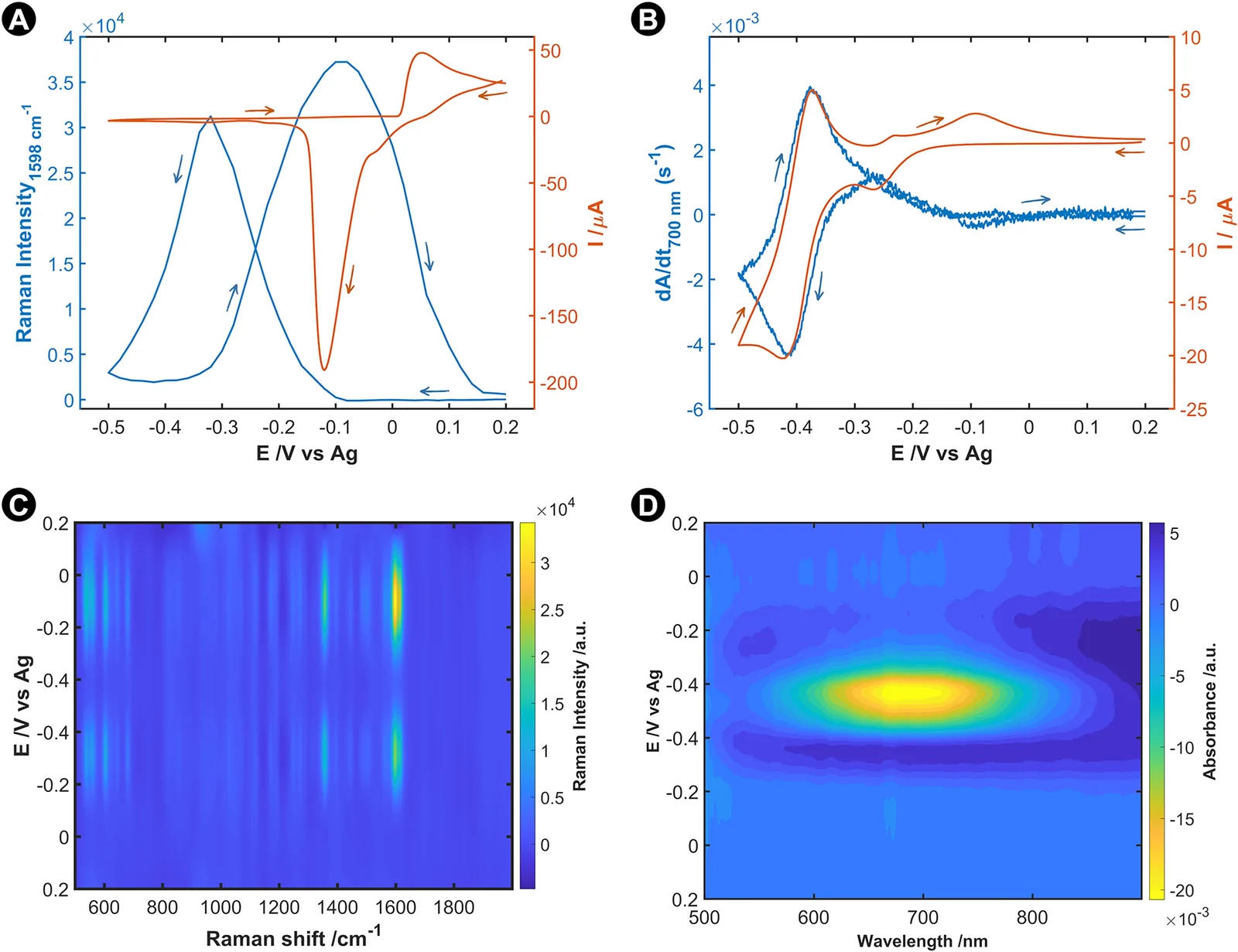
(A) CV (orange line) and voltaRamangram at 1598 cm^–1^ (blue line) for an 18.5 nM PYO sample in BRB (pH = 6), 0.1 M LiClO_4_, and 10 mM KCl using an Ag-SPE. The CV started at +0.20 V in the cathodic direction down to the vertex potentials (−0.50 V) at a scan rate of 0.02 V·s^–1^, after applying an initial potential of +0.20 V for 5 s. (B) CV (orange line) and derivative voltabsorptogram (blue line) at 700 nm for the TR-UV/vis-SEC experiment performed in a solution containing 555 μM PYO in BRB (pH = 6) and 0.1 M LiClO_4_ using an Au-SPE. (C, D) Contour plots showing the evolution of the PYO Raman spectra (500–2000 cm^–1^) and the evolution of the absorption spectra (500–900 nm), respectively, with the applied potential during the CV.

Figure 1A shows the CV together with the evolution of the Raman intensity of the most characteristic PYO band, located at 1598 cm^–1^, as a function of the applied potential. This representation, denoted as the VoltaRamangram^42,59^ at 1598 cm^–1^, plots the intensity of a selected Raman band during the electrochemical scan, allowing to directly correlate the spectroscopic response with the applied potential. This characteristic Raman band of PYO corresponds to the C–C stretch vibration,^56^ which shows the highest Raman intensity in the spectrum. One spectrum of PYO on the prepared SERS substrate is shown in Figure S4A, and the assignment of Raman bands for pyocyanin is summarized in Table S1.^56^ As can be observed in the CV (Figure 1A, orange line), initially, the Ag surface was oxidized to yield AgCl nanocrystals (NCs) on the electrode surface, as can be deduced from the anodic current observed. A reduction peak, related to the reduction of AgCl NCs to generate Ag NPs on the electrode surface was observed at −0.13 V. This peak reached an almost zero current when the deposited AgCl was fully reduced on the WE surface. In the anodic scan, an oxidation peak evolved at +0.05 V, which was related to the oxidation of the Ag NPs deposited on the electrode to generate AgCl NCs. This typical CV does not yield enough information about all the changes taking place on the WE surface, but the voltaRamangram at 1598 cm^–1^ illustrates the complexity of the electrode process (Figure 1A, blue line).

As can be observed, the voltaRamangram at 1598 cm^–1^ starts to evolve at −0.10 V, when the AgCl NCs deposited on the electrode are reduced to Ag NPs, enhancing the Raman intensity and giving a typical SERS signal. The Raman intensity increased until a potential of −0.30 V was applied, at which point a sudden drop was observed.

The enhancement of the Raman signal was related to the complete reduction of AgCl NCs to generate Ag NPs. However, the decrease in Raman intensity cannot be fully explained by either the voltaRamangram or the CV. During the backward scan, the Raman signal was negligible between the vertex potentials of −0.50 V and −0.30 V; however, the Raman signal was enhanced again from −0.30 up to −0.10 V, yielding the highest Raman signal in the experiment at −0.10 V. From this potential onward, the Raman signal decreased again due to the oxidation of the Ag NPs to yield AgCl NCs, losing the SERS effect. Notably, all the Raman bands related to the PYO evolves in the same way, as can be seen in the contour plot of the Raman-SEC experiment, Figure 1C, which provides the evolution of the whole Raman spectrum during the experiment. The SERS spectrum is very similar to the normal Raman spectrum of PYO, Figure S4B. The SERS performance factor of our SERS substrate has been calculated according with the methodology proposed by Ren et al.^60^ obtaining a value of 3.7·10^6^. Control experiments in the absence of PYO showed a background signal where no evolution of the band at 1598 cm^–1^ was detected, Figure S5A, confirming that the signal obtained in the presence of PYO is related to this molecule.

The explanation of the evolution of the Raman signal is not obvious from the recorded signals (Raman spectra and CV) and can only be partially explained. TR-UV/vis-SEC can help to fully understand all the processes taking place on the electrode surface. A similar experiment was performed using TR-UV/vis-SEC in the normal configuration using Au-SPE to follow the redox behavior of PYO. In the TR-UV/vis-SEC experiments, the absorption spectrum of the initial solution was taken as a reference spectrum, so that a positive change in absorbance was related to the generation of a product, while a negative change in absorbance is related to the consumption of the reactant, PYO. Figure 1B,1D summarize the results obtained during the TR-UV/vis-SEC experiment. As expected, the CV (Figure 1B, orange line) is completely different from that observed in Figure 1A because at Au-SPE there is no redox processes that modify the WE surface at these potentials. A small reduction peak was observed at approximately −0.25 V, which was also observed in an experiment carried out in the absence of PYO (Figure S5B). From −0.30 V to the vertex potential, a clear reversible electrochemical reaction was observed, with a formal potential of −0.40 V vs Ag/AgCl. Based on the literature,^34,39,58^ and considering that the experiment was performed at pH 6, above the p*K*_a_ of PYO (∼4.9),^58^ this process can be assigned to the two-electron/two-proton reduction of oxidized PYO to its reduced form, PYO_red_. Upon the reverse scan, PYO_red_ is reoxidized back to PYO.^58^

The spectroscopic signal obtained concomitantly with the electrochemical signal confirms that this electrochemical process is related to the PYO, as can be observed in both the evolution of the absorption spectra, Figure 1D, and the derivative voltabsorptogram at 700 nm (Figure 1B, blue line). A band around 700 nm, related to the π→π* transition between HOMO and LUMO of the PYO, decreases from −0.30 V to −0.50 V indicating that PYO is being reduced, which in the backward scan is subsequently oxidized to generate again PYO.^45,47^ This band is responsible for the resonance Raman process, which, combined with the enhancement of the Ag NPs, yields a SERRS behavior in the Raman experiment. The derivative of the voltabsorptogram is the spectroscopic response that can be considered similar to the CV for simple electron transfer processes controlled by diffusion.^61^ The evolution of the cyclic voltabsorptogram at 700 nm is plotted in Figure S5C (blue line), and its derivative is shown in Figure 1B (blue line). The high similarity between the derivative voltabsorptogram at 700 nm and the CV in the potential region below −0.30 V confirms the electrochemical process that this molecule undergoes. The same redox process occurs during TR-EC-SERS, but it is not electrochemically detected because of the low concentration of PYO.

After the TR-UV/vis-SEC experiment, the voltaRamangram at 1598 cm^–1^ can be fully explained. In the potential window in which PYO is in its oxidized form, the electrochemical surface-enhanced Raman resonance scattering (EC-SERRS) process is observed because the laser excitation wavelength of 785 nm falls within the spectral region of the PYO absorption band. Below −0.30 V, the Raman signal decreased due to the electrochemical reduction of PYO. Reduced PYO does not absorb in the visible region around 700 nm; therefore, resonance Raman enhancement is not favored, leading to a decrease in the SERRS signal. However, when PYO is regenerated during the oxidation process in the backward scan, the SERRS signal increases again.

This behavior is interesting for the detection of this molecule because TR-EC-SERRS provides a double-fingerprint of the molecule. On the one hand, the Raman spectrum is the typical fingerprint of the molecule; on the other hand, the evolution of the Raman signal provides a second fingerprint of PYO because it shows a characteristic ‘butterfly’ shape (blue line in Figure 1A), that conjugates the reduction process and the resonance Raman effect. This characteristic evolution of the Raman signal can be considered a second fingerprint because this signal is unique to PYO, as very few, if any, molecules with SERS enhancement are reversibly reduced at a specific potential, and the Raman shifts are associated with PYO. A CV cannot be considered a fingerprint because many compounds can be oxidized/reduced at very similar potentials. However, a cyclic voltaRamangram contains molecular specificity because of the Raman spectra together with electrochemical specificity because of the oxidation/reduction process. It is very difficult to find two molecules that exhibit a similar Raman spectra and similar oxidation/reduction potentials. For example, assuming that two molecules are oxidized/reduced at the same potential, it rather unlikely that more than one Raman band evolves at the same Raman shift. On the contrary, assuming that the spectra are very similar, it is quite difficult for two molecules to be oxidized/reduced at the same potential. In our case, phenazines show similar structures and, therefore, similar Raman spectra, but they are not equal and these molecules are not reduced at the same potential. Table S2 shows the formal potentials of several phenazines and, as can be observed, there is huge difference on the values of these potentials.

This experiment not only demonstrates the capability of SEC techniques to understand the electrode process but also demonstrates the capability of SEC techniques to improve the selectivity of individual analytical methods.

### Working Electrode Surface Characterization

Scanning electrochemical microscopy experiments were performed to characterize the nanostructures generated during the different stages of SERRS substrate generation. SEM images were obtained at the numbered positions in Figure S3A: (1) Pristine electrode, Figure S6A,B (2) AgCl NCs generated on the electrode surface after the pretreatment step, Figure 2A,B, (3) Ag NPs generated on the electrode surface at −0.30 V, Figure 2C,D, and (4) Ag NPs generated on the electrode surface at −0.50 V, Figure S6C,D.

**Figure 2:**
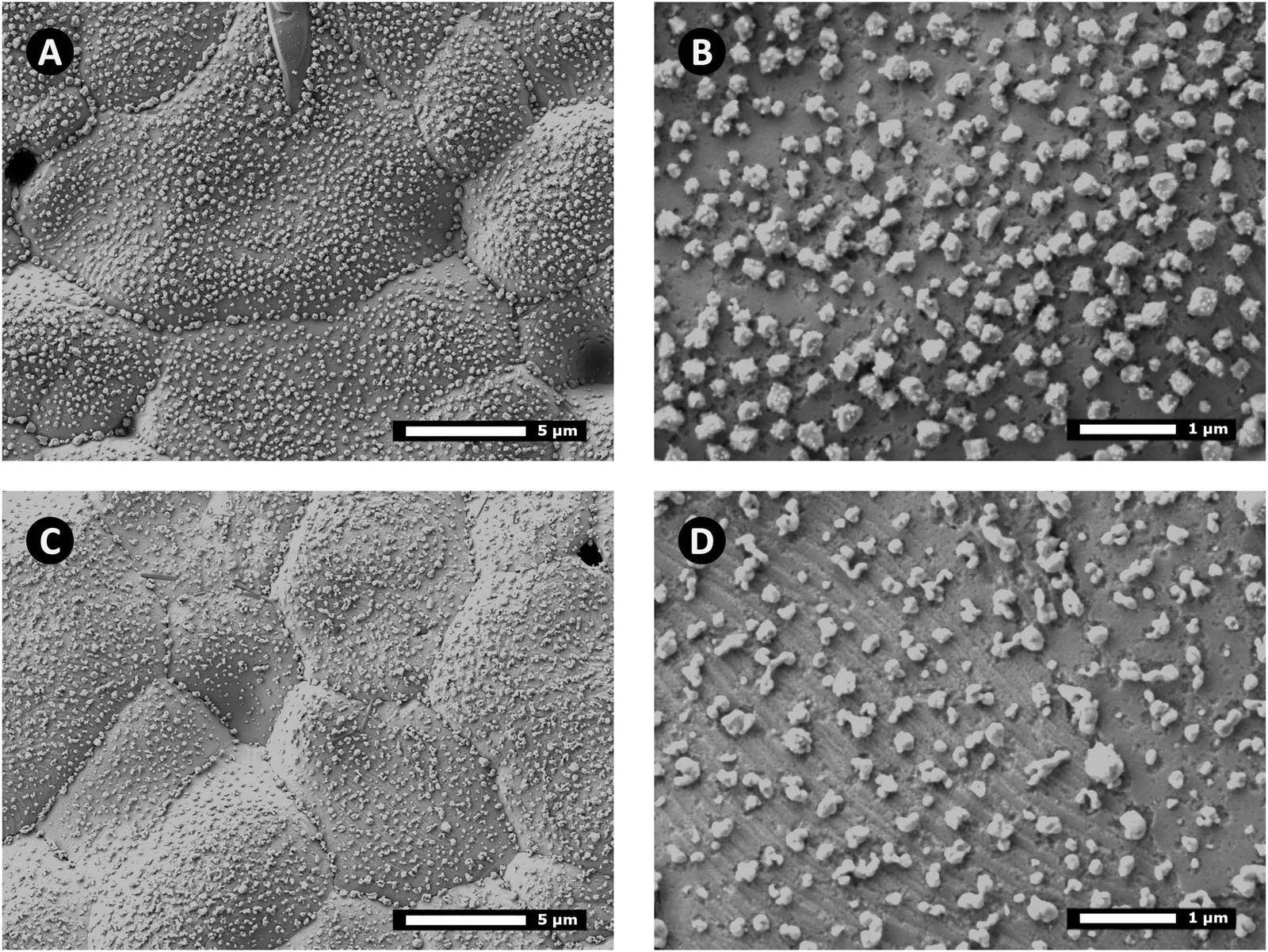
SEM images of the Ag-SPE surface during the SEC experiment. (A, B) SEM images at different magnifications of the Ag-SPE surface acquired after the conditioning step and prior to the measurement step. (C, D) SEM images at different magnifications of the Ag-SPE surface acquired at – 0.30 V during the LSV measurement.

As shown in Figure 2A,B, quasi-cubic AgCl NCs were formed on the electrode surface at the end of the pretreatment step. These NCs served as seeds to generate Ag NPs during the reduction scan, resulting in quasi-spherical Ag NPs (Figure 2C,D). These Ag NPs enhanced the Raman signal, yielding excellent SERRS performance. As expected, a small decrease in the diameter of the NPs was observed because of the change in the crystal structure from AgCl to Ag, losing the chloride in the crystal lattice. It is noteworthy that the size dispersion is lower for AgCl NCs, compared with the higher size dispersion observed for Ag NPs, Figures 2 and S7, which are agglomerated in some cases, potentially generating the typical hot-spots that enhance the Raman intensity.

While a wider size distribution of NPs can be a problem in confocal Raman spectroscopy to obtain reproducible results, in low-resolution Raman spectroscopy, the laser spot size (approximately 200 μm) is much larger than that of confocal Raman instruments, providing much more representative information on the whole surface than confocal Raman spectroscopy, which is demonstrated by the good reproducibility of the measurements under the same conditions in different Ag SPEs (see below for the quantitative results).

Once Ag NPs were formed, there was no change in shape or size until the end of the experiment, as can be deduced from the comparison of Figures 2C and S6C. A more straightforward comparison can be made in Figures 2D and S6D, which show a magnification of the nanostructures formed at −0.30 V and −0.50 V, respectively. Therefore, this confirms that the decrease in the Raman signal is not related to changes in the nanostructure responsible for the SERS enhancement but to the electrochemical reduction of PYO.

### Effect of pH in the SERS Response

As shown in the SEC analysis of PYO, a well-defined SERRS spectrum of this molecule can be obtained using Ag NPs as the SERS substrate. Because the aim of this study was to detect PYO in highly complex matrices of different natures (direct bacterial culture, serum, saliva, urine, and tap water), it is critical to operate in a buffered medium. Thus, the pH variations introduced by the sample itself can be minimized. Otherwise, the generation of NPs and, consequently, the sensitivity and reproducibility of the proposed method could be affected. Therefore, a pH study was conducted to evaluate the effect of pH on the measurements in terms of sensitivity and reproducibility (Figure S8). As can be seen, although experiments at pH = 4 showed higher sensitivity, the reproducibility was poor for the requirements of this work. This poorer precision could be related to the proximity of this pH to the p*K*_a_ of PYO (∼4.9).^58^ In contrast, measurements at pH = 6 provided a more reliable balance between sensitivity and reproducibility, yielding a more consistent response. Accordingly, a pH = 6 was selected as the working condition for subsequent experiments.

### Quantitative Analysis

A calibration curve was generated to demonstrate the capability of TR-EC-SERRS to determine PYO at low concentrations. For this purpose, seven standard solutions between 0.37 nM and 101.75 nM were prepared in an aqueous solution containing BRB (pH = 6), 0.1 M LiClO_4_, and 10 mM KCl as the supporting electrolyte. Analytical measurements were performed using LSV, scanning the potential between +0.20 V and −0.50 V, according to the protocol described in Figure S3B. CV experiments usually provide more sensitive but less reproducible voltaRamangrams in the backward scan. However, more reproducible signals are registered by limiting the analysis to the cathodic scan using LSV. In SERS analyses, a fixed integration time is typically applied to all samples; however, this often causes the photon detector to saturate, resulting in a very narrow linear concentration range. To overcome this limitation and extend the linear dynamic range, Raman spectra were acquired using different integration times. This approach yielded highly reliable quantitative results across nearly 3 orders of magnitude of PYO concentration. The integration times used for each PYO concentration are summarized in Table S3, with the longest integration time (4 s) applied to the most diluted sample and the shortest integration time (100 ms) applied to the most concentrated one. In this case, the electrode was pretreated using the protocol described in Figure S2A.

To assess the changes in the voltaRamangrams at 1598 cm^–1^ as a function of the PYO concentration, it was necessary to normalize the Raman signal by the integration time used in each experiment (Table S3). As shown in Figure 3A, the normalized voltaRamangrams at 1598 cm^–1^ for the seven PYO standards exhibited a clear increase in signal intensity with increasing concentration. The inset in Figure 3A shows the SERRS response at the lowest PYO concentrations in the pM range. To improve the sensitivity of the PYO determination using the Raman signal, the cumulative sum of the Raman intensity at 1598 cm^–1^ was used throughout the entire LSV experiment (35 s). Figure 3B shows the resulting calibration curve, which includes triplicate measurements of the standard samples (orange dots) and the test sample (yellow dot).

**Figure 3:**
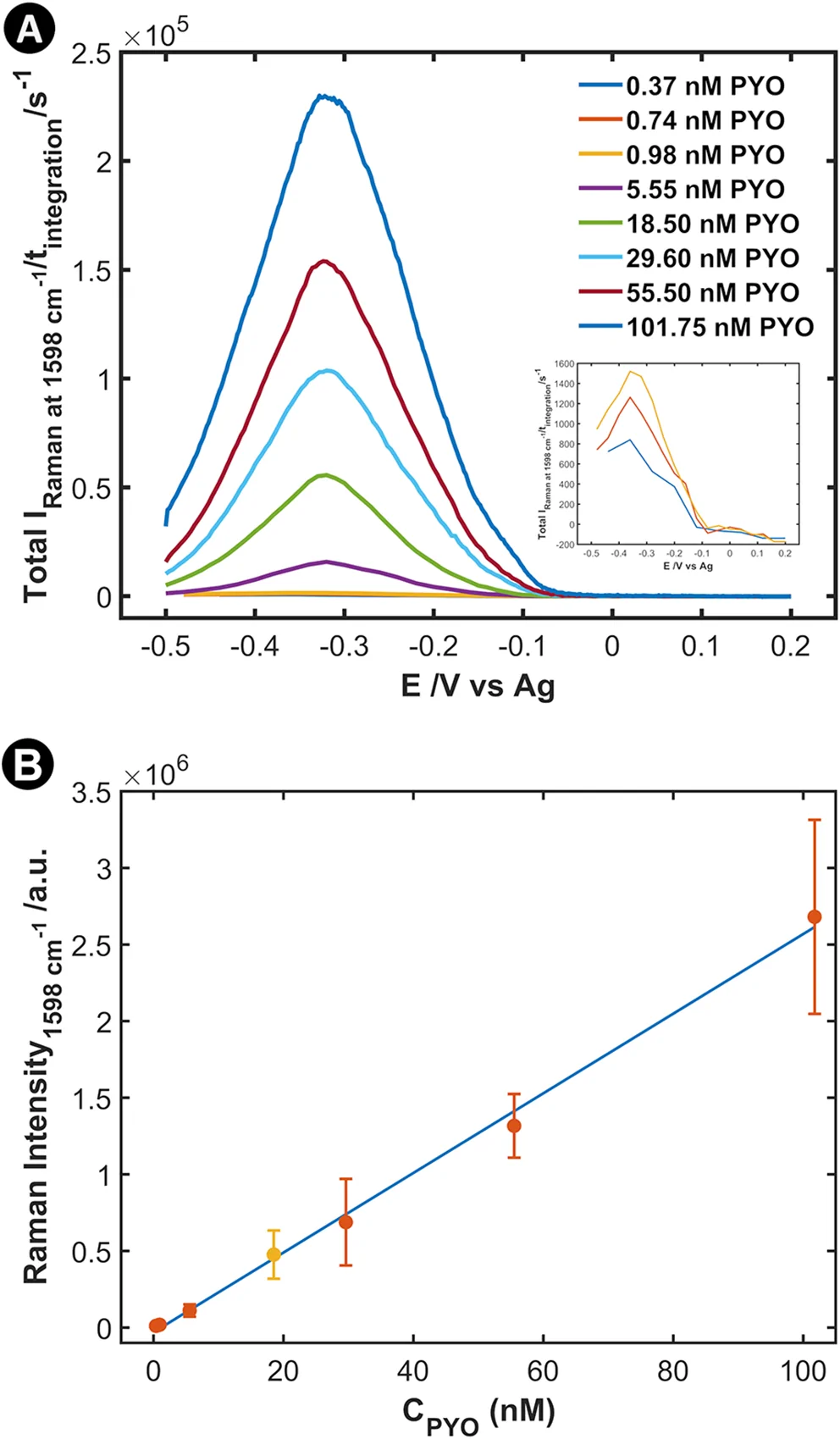
Normalized voltaRamangrams at 1598 cm^–1^ by the integration time for PYO concentrations in the range from 370 pM to 101.75 nM in 0.4 M Britton-Robinson buffer (BRB, pH = 6), 0.1 M LiClO_4_, and 10 mM KCl. (B) Calibration curve obtained from the cumulative sum of the Raman intensity at 1598 cm^–1^ recorded during the entire LSV experiment (35 s).

As can be observed, the method exhibits good linearity (*I*_R_, 1586 cm^–1^ = 2.598·10^4^·C_PYO_ - 2.966·10^4^, *R* ^2^ = 0.997, *S*_*yx*_ = 6.087·10^4^), evidenced by a determination coefficient (*R* ^2^) close to 1 and a low standard deviation of the residuals (*S*_*yx*_).

Above 100 nM, the linear response is lost, resulting in less reproducible results. The estimated limit of detection (LOD), obtained from the calibration curve using the four most diluted samples, was 299.8 pM, defined as 3.3·*S*_*yx*_/*m*, where *m* is the slope of the calibration curve (I_R_, 1586 cm^–1^ = 1.966·10^4^·C_PYO_ – 0.236·10^4^, *R* ^2^ = 0.999, *S*_*yx*_ = 1.786·10^3^). As shown in the inset of Figure 3A, PYO was clearly detected using this methodology at pM concentrations. The prediction capability of the method was verified by measuring a test sample fortified in deionized water at 18.5 nM PYO, yielding a recovery of 105%. Moreover, the method demonstrated good reproducibility for SERRS measurements, with a relative standard deviation of the concentration (%RSD) of 8.6 (*n* = 4), which is much lower than the commonly accepted 20% threshold for SERS analysis.^62^

### Interfering Compounds

As discussed above, very low concentrations of PYO can be detected in simple samples. However, the key point of this work is the detection of PYO in the presence of other molecules that could interfere with the Raman signal. On the one hand, PYO exhibits a very characteristic SERRS spectrum that can be considered a molecular fingerprint. On the other hand, the voltaRamangrams recorded for the different PYO Raman bands displayed a distinctive ‘butterfly’-shaped pattern, due to the electrochemical reduction of the molecule and the consequent loss of the Resonance Raman enhancement. This characteristic spectroelectrochemical behavior provides a second fingerprint for the unambiguous identification of PYO in complex mixtures of analytes. Therefore, TR-EC-SERRS experiments supply a double-fingerprint which is a double-check of the presence of this target molecule, offering a significant advantage over other analytical techniques. To demonstrate the detection capability of this molecule using TR-EC-SERRS, experiments in the presence of adenine (AD) and folic acid (FA) were performed.

These two molecules were selected because adenine is a typical molecule detected by SERS that can be found in many biological samples, with the main Raman band peaking at 733 cm^–1^. Moreover, the spectrum was very similar to that of adenosine, which is often present in bacterial culture media. Folic acid was selected because this molecule exhibits a Raman band at a very close position, 1561 cm^–1^, to the main peak of PYO, 1598 cm^–1^.

Figure 4A shows the Raman spectra of the three molecules (PYO, folic acid, and adenine) and mixtures of folic acid/PYO and adenine/PYO. The characteristic bands of each molecule are marked with a rectangular band of the same color as the molecular spectrum. The characteristic bands of the molecules were clearly defined, and in the case of the folic acid/PYO mixture, a high overlap of the main Raman band was clearly observed. Figure 4B,C show the voltaRamangrams at 1598 cm^–1^ of the folic acid/PYO and adenine/PYO mixtures, respectively. Figure S9 depicts the voltaRamangrams at the most representative Raman shift for each pure molecule. The voltaRamangram of folic acid at 1561 cm^–1^ (Figure S9B) shows an increase in the Raman intensity from 0 V to −0.30 V, reaching a steady value from −0.30 V onward. In the backward anodic scan, the signal decreased from −0.10 V downward when the Ag NPs were oxidized to AgCl NCs. For adenine, the Raman intensity at 733 cm^–1^ (Figure S9C) increased at cathodic potentials below −0.10 V, which was ascribed to the adsorption of the molecule onto the Ag NPs. During the subsequent anodic scan, the signal decreased as adenine desorbed from the NPs surface. From 0 V downward, the oxidation of Ag NPs to generate AgCl NCs induced an even more pronounced decrease in the Raman signal. Finally, the voltaRamangrams of PYO at the three characteristic bands of the spectra show the previously described ‘butterfly’-shaped pattern, (Figure S9A).

**Figure 4:**
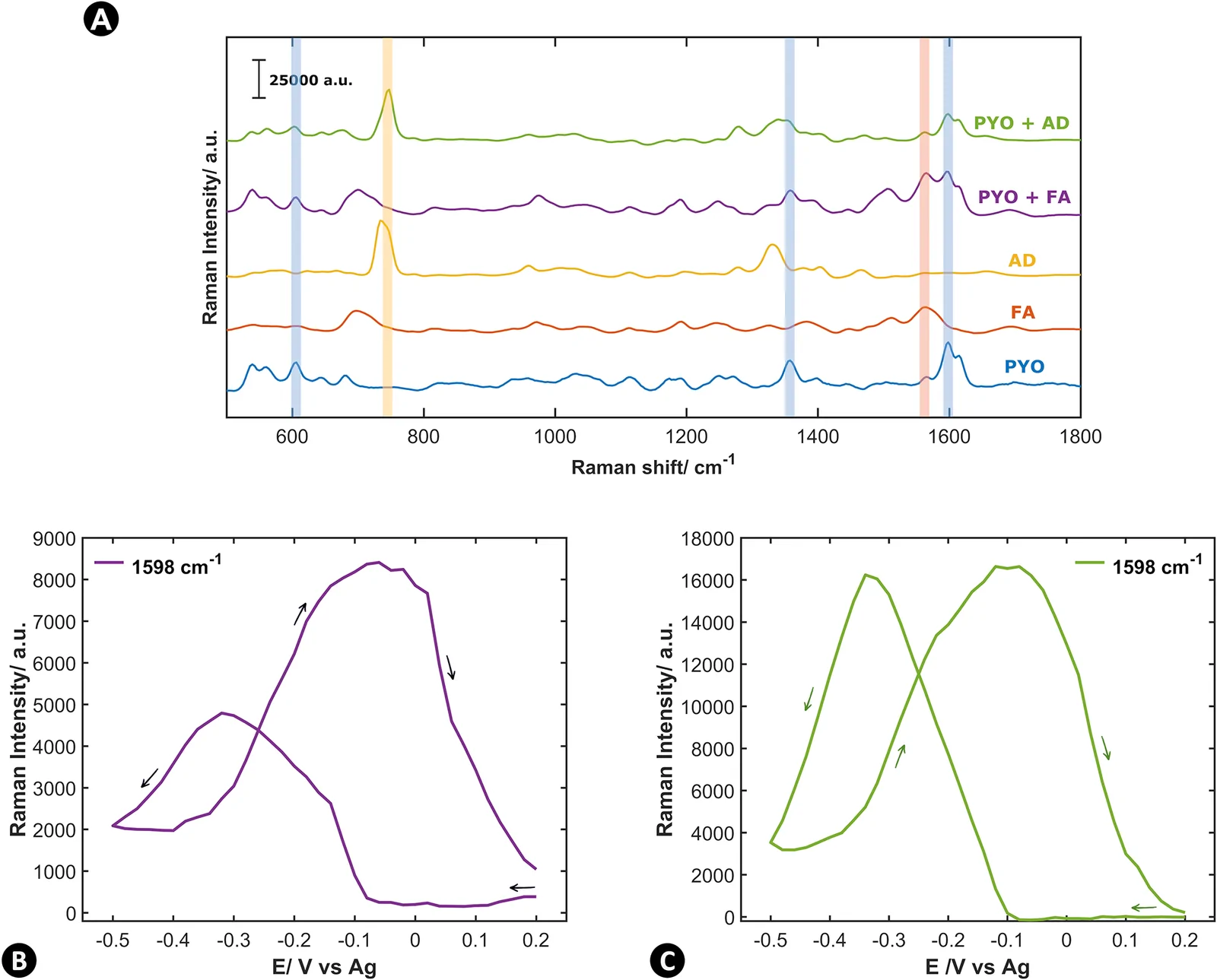
(A) Raman spectra of PYO (5 nM, blue), folic acid (5 μM, orange), adenine (5 μM, yellow), a mixture of folic acid (5 μM)/PYO (5 nM) (purple), and a mixture of adenine (5 μM)/PYO (5 nM) (green) during the TR-EC-SERS experiments. The characteristic bands of each molecule are indicated by the shaded regions of the corresponding color. VoltaRamangrams recorded at 1598 cm^–1^ for (B) the folic acid/PYO mixture and (C) the adenine/PYO mixture.

When the samples contained only one of these molecules, the distinctive ‘butterfly’ fingerprint was only observed for PYO. However, in the EC-SERRS experiments performed for the mixtures, both specific fingerprints of PYO were still clearly observed: (a) the characteristic SERRS spectrum of PYO and (b) the unique ‘butterfly’-shaped profile of its voltaRamangrams (Figure 4B,C). Moreover, the ‘butterfly’ shape is also observed at the less intense characteristic bands of PYO, 1357 cm^–1^ and 605 cm^–1^, in the mixtures with folic acid (Figure S10A,B) and with adenine (Figure S10E,F). Figure S10C,D show the profiles of the characteristic bands of folic acid and adenine, respectively, in the mixtures, demonstrating that their behavior was similar to that observed for the pure compounds (Figure S9B,C, respectively).

Based on these experiments, it can be concluded that the TR-EC-SERRS methodology enables the acquisition of a double fingerprint for PYO, facilitating its unequivocal detection even in the presence of interfering compounds with Raman bands located in similar positions in the spectrum.

### Analysis of PYO in Complex Matrices

Four different complex matrices, tap water, urine, saliva, and serum, were selected to demonstrate the capability of detecting PYO using TR-EC-SERRS. LSV experiments were performed at different PYO concentrations using the protocol described in Figure S2A. In all cases, the samples were fortified with PYO at nM concentrations, between 1 and 20 nM in tap water and between 200 nM and 1.5 μM in the other three matrices. The samples were diluted to 40% in the case of tap water and to 1% in the other matrices, to minimize the matrix effect and to prepare them in the analysis medium, BRB (pH = 6), 0.1 M LiClO_4_ and 10 mM KCl. Interfering compounds predominantly affect the electrochemical process involved in the preparation of Ag-SERS substrates. Insets in Figure 5 show the voltaRamangrams at 1598 cm^–1^ and the characteristic spectra of PYO in the four complex matrices tested at different concentrations. Both signals, spectra and voltaRamangrams at 1598 cm^–1^, provide unequivocal proof of the presence of PYO in the samples. Blank measurements, performed using only the electrolyte medium, were included to demonstrate that any spectral or voltaRamangram feature observed arose only from PYO.

**Figure 5:**
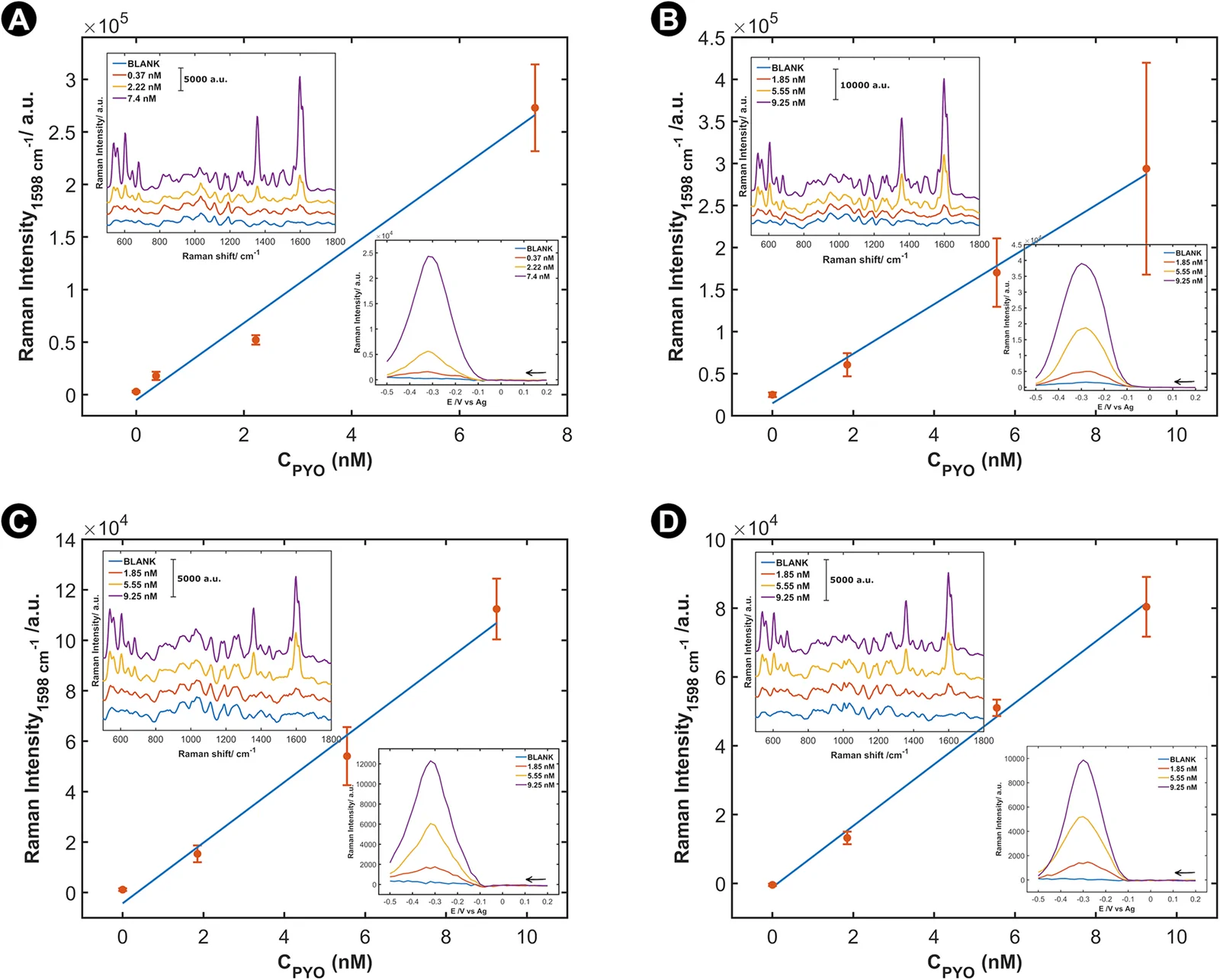
Linear regression of the cumulative sum of the Raman intensity at 1598 cm^–1^ along the whole experiment as a function of the PYO concentration in four complex matrices: (A) tap water, (B) urine, (C) saliva, and (D) serum. The upper insets display the Raman spectra recorded at – 0.30 V for the different concentrations, whereas the lower insets depict the voltaRamangrams at 1598 cm^–1^ for each concentration tested. Blank measurements were included in all cases to confirm the absence of characteristic PYO in the spectral and voltaRamangram features.

The calibration curves for each matrix are shown in Figure 5. The figures of merit corresponding to each regression for the different matrices are listed in Table S4. The Raman intensity at 1598 cm^–1^ was integrated for the full 35 s experiment to construct the calibration curves. In all cases, the cumulative Raman intensity at 1598 cm^–1^ increased linearly with the PYO concentration. The LOD obtained for PYO were 4.45 nM in tap water, 134 nM in urine, 227 nM in saliva, and 95 nM in serum. The high sensitivity, combined with the significant qualitative information provided by double-fingerprinting, renders the TR-EC-SERRS methodology an invaluable tool for detecting hazardous molecules across diverse environments. This capability is particularly crucial for the clinical analysis of infected patients. The very low detection limits achieved in these complex samples are below the typical PYO concentrations reported for real samples,^27,63–65^ which supports the potential application of the TR-EC-SERS methodology in clinical analysis.

### Monitoring of PYO Secretion in Culture Media

After demonstrating the performance of TR-EC-SERRS in the detection of molecules in the absence of bacteria, experiments were performed in a culture medium of *PA* to demonstrate the usefulness of the new methodology in registering the trend in the generation of PYO during the strains culture. In accordance with the cultivation protocol outlined in the Experimental Section, aliquots were extracted each hour from the initial time point up to 8 h for subsequent analysis. In a 1.5 mL microcentrifuge tube, 0.5 mL of ether was added to 0.5 mL of each sample, Figure S2A,B, and the two solutions were mixed by manually shaking the closed tube for 1 min. After ether evaporation, which required approximately 30 min at room temperature, Figure S2C, an aliquot of the resulting extract was transferred to a suitable medium for TR-EC-SERRS measurements at a 1:100 dilution. The use of ether offers the advantage of *PA* inactivation, as this organic solvent can compromise bacterial integrity and suppress bacterial activity. Cultures were performed with a solution containing bacteria treated with ether, yielding negative results for the presence of *PA* in all cases. Figure S2D shows a culture plate presenting bacterial growth from an untreated solution with ether, and Figure S2E shows the absence of bacterial growth when the solution is treated with ether.

The time evolution of the SERRS spectra during the culture growth is shown in Figure 6A. From these spectra, it can be deduced that PYO is secreted in the fifth hour since the Raman band at 1598 cm^–1^ starts to be observed. From this point onward, other characteristic Raman bands ascribed to PYO are more clearly observed. In fact, in the spectrum obtained in the fourth hour, some spectral features of PYO could be considered. A SERRS spectrum alone is not enough to confirm the presence of PYO in the sample, but TR-EC-SERRS also provides the evolution of the Raman bands with potential. Figure S11A,B show the voltaRamangrams at 1598 cm^–1^ obtained for the samples tested at different culture times.

**Figure 6:**
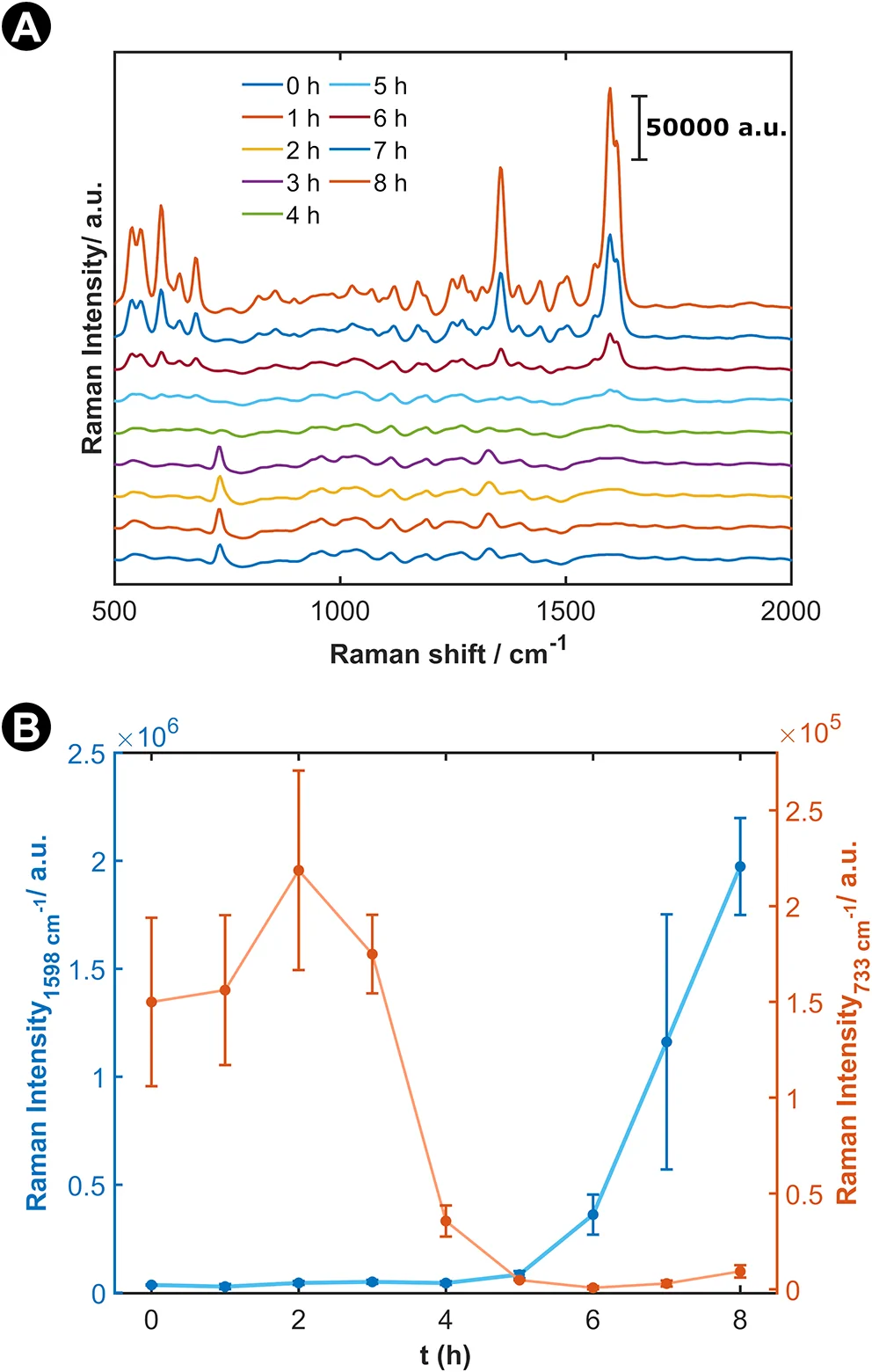
(A) SERRS spectra obtained by the cumulative sum of the Raman intensity during the 35 s TR-EC-SERRS experiment for aliquots collected from the microbial culture at different culture times (0–8 h). (B) Evolution of the Raman intensity at 733 cm^–1^ and 1598 cm^–1^ as a function of time (0–8 h).

As can be seen, the typical shape of the voltaRamangram of PYO is detected from the fourth hour to the eighth hour. Therefore, it can be concluded that PYO is secreted by *PA* during this early stage. The evolution of OD_600_ measured before sample analysis, Figure S11C, demonstrated that *PA* was growing in the culture medium, but PYO was only detected from the fourth-5th hour onward. False negatives at shorter times could be possible because of the detection capability of the method, however, the generation of PYO is not expected at these earlier stages of growth.

A detailed analysis of the Raman spectra recorded during the first 3 h of culture revealed the presence of additional molecules. In particular, adenosine was identified by its characteristic Raman band at 733 cm^–1^, which could be attributed to the presence of this molecule in the MH culture medium. Adenosine remained relatively stable during the first 3 h, began to decrease in the fourth hour, and was no longer detected from the fifth hour onward. As illustrated in Figure 6B, the decrease in the adenosine Raman intensity at 733 cm^–1^ correlates with the increase in the PYO Raman intensity at 1598 cm^–1^. These results indicate that PYO secretion could be associated with a decrease in adenosine levels in the MH culture medium, probably indicating the use of adenosine by *PA* to support its metabolic activity.^66^

These results indicated that TR-EC-SERRS can monitor the temporal evolution of PYO secretion in a culture medium in which *PA* is growing. Moreover, this technique provides information on additional compounds present in the culture medium, allowing for a more comprehensive understanding of the biochemical processes occurring during the growth of *PA* bacteria.

## Conclusions

A new methodology for the detection of PYO as a biomarker of *PA* based on TR-EC-SERRS has been developed. This methodology provides a double fingerprint of the molecule, not only from the characteristic spectrum of the molecule but also from the characteristic voltaRamangram due to the reversible electrochemical properties of PYO. Particularly, by using CV, a ‘butterfly’ shape of the voltaRamangram was obtained. The EC-SERRS response can be used to quantify PYO at low concentrations, reaching a LOD of 299.8 pM in synthetic solutions. PYO can also be detected in complex matrices such as tap water, urine, saliva and serum, obtaining unequivocal signals of the presence of PYO in the nM range of concentration with a simple sample pretreatment based only on a dilution step. In the case of bacterial analysis, a simple and fast sample pretreatment based on the addition of ether to the culture medium has been proposed.

## Supplementary Material



## Notes

Description of *Pseudomonas aeruginosa* strain and culture conditions; sample preparation by ether treatment; EC-SERS measurements; assignment of Raman bands for PYO; Raman and UV/vis spectroelectrochemical behavior of PYO; surface characterization; particle size distribution; pH study; optimization of SERRS integration time for the calibration curve; interference studies; linear regression curves for complex matrices; pyocyanin secretion analysis (PDF)
