# Supplementary material for: Time-Resolved Electrochemical Surface-Enhanced Resonance Raman Spectroscopy Double-Fingerprint for the Detection of Pseudomonas aeruginosa
Source: ACS Meas Sci Au. 2026 Jul 30;6(4):1326–38. doi: 10.1021/acsmeasuresciau.6c00144 (PMC13495737; doi:10.1021/acsmeasuresciau.6c00144)
Supplement: Supplementary file 1 [file tg6c00144_si_001.pdf]

SUPPORTING INFORMATION:

Time-Resolved Electrochemical Surface-Enhanced Resonance Raman Spectroscopy Double-Fingerprint for the Detection of *Pseudomonas aeruginosa*

Luis Romay[a], *Esther Carcelen*[a], *Sandra de la Parra*[b], *Sara Collado*[b], *Rocio Barros*[b], *Carlos Rumbo*[b], *Aranzazu Heras**[a], *Alvaro Colina**[a]

a Department of Chemistry, Universidad de Burgos. Plaza Misael Bañuelos s/n, E-09001 Burgos. Spain.

b International Research Center in Critical Raw Materials for Advanced Industrial Technologies-ICCRAM, Universidad de Burgos, Plaza Misael Bañuelos s/n, E-09001 Burgos. Spain

Table of contents

1. Bacterial strain and culture conditions. 3
2. Sample preparation. Ether treatment. 4
3. EC-SERS measurements. 5
4. Assignment of Raman bands for pyocyanin. 7
5. Phenazine derivatives formal potentials. 8
6. Raman and UV/Vis spectroelectrochemical behavior of pyocyanin. 9
7. Surface characterization. 10

8. Particle size distribution. 11
9. pH Study. 11
10. SERRS Integration time for the calibration curve. 12
11. Interference study (I). 12
12. Interference study (II). 13
13. Linear regression curves for complex matrices. 14
14. Pyocyanin secretion. 14
15. References. 15

1. Bacterial strain and culture conditions.

Throughout this work, a standardized culture protocol was used to obtain cultures at a controlled optical density (OD_{600} = 0.04). The procedure started with a frozen stock of *Pseudomonas aeruginosa* (*PA*), from which a small amount of biomass was collected and streaked onto Mueller–Hinton (MH) agar (first plating). The plates were incubated for 24 h at 37 °C. After incubation, bacterial growth was observed and a new MH agar plate was inoculated by re-streaking from the first plate (second plating). The second plate was incubated under the same conditions for 24 h at 37 °C. Next, a single colony was transferred to MH broth in a sterile conical tube and cultivated for 24 h at 37 °C with shaking at 180 rpm (pre-inoculum). The OD_{600} of the pre-inoculum was then measured, and the culture was diluted with fresh MH broth to an OD_{600} of 0.04. This diluted culture was incubated in MH broth at 37 °C and 180 rpm in a suitable vessel, and aliquots were withdrawn at the required time points for subsequent analysis.

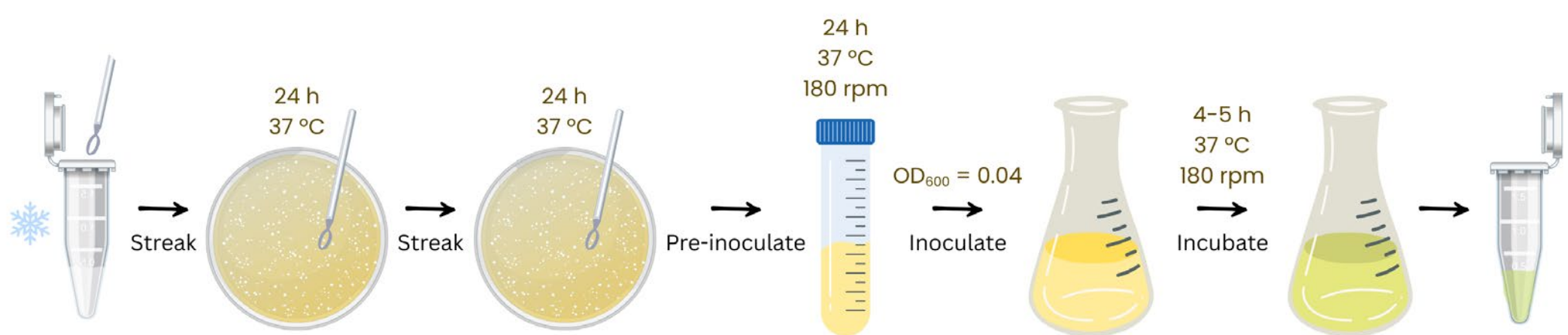

Figure S1. Schematic representation of the culture and sampling procedure used for *Pseudomonas aeruginosa* PAO1.

2. Sample preparation. Ether treatment.

All biological samples were handled and cultured in a biological laboratory equipped with appropriate facilities and safety measures for working with *PA*. After the bacterial culture and growth steps were complete, a final aliquot was collected according to the protocol described in the main text section. Specifically, 0.5 mL of the resulting suspension, which contained an unknown bacterial load, was transferred to a 1.5 mL microcentrifuge tube. The samples were then treated with ether at a 1:1 (v/v) ratio, directly in the same tube. The contents were vortex-mixed vigorously with the tube closed, after which the tube was opened and left in a fume hood until complete solvent evaporation.

Control experiments were repeated to confirm that this procedure effectively inactivated the bacteria. These verification assays are summarized in Figure S2.

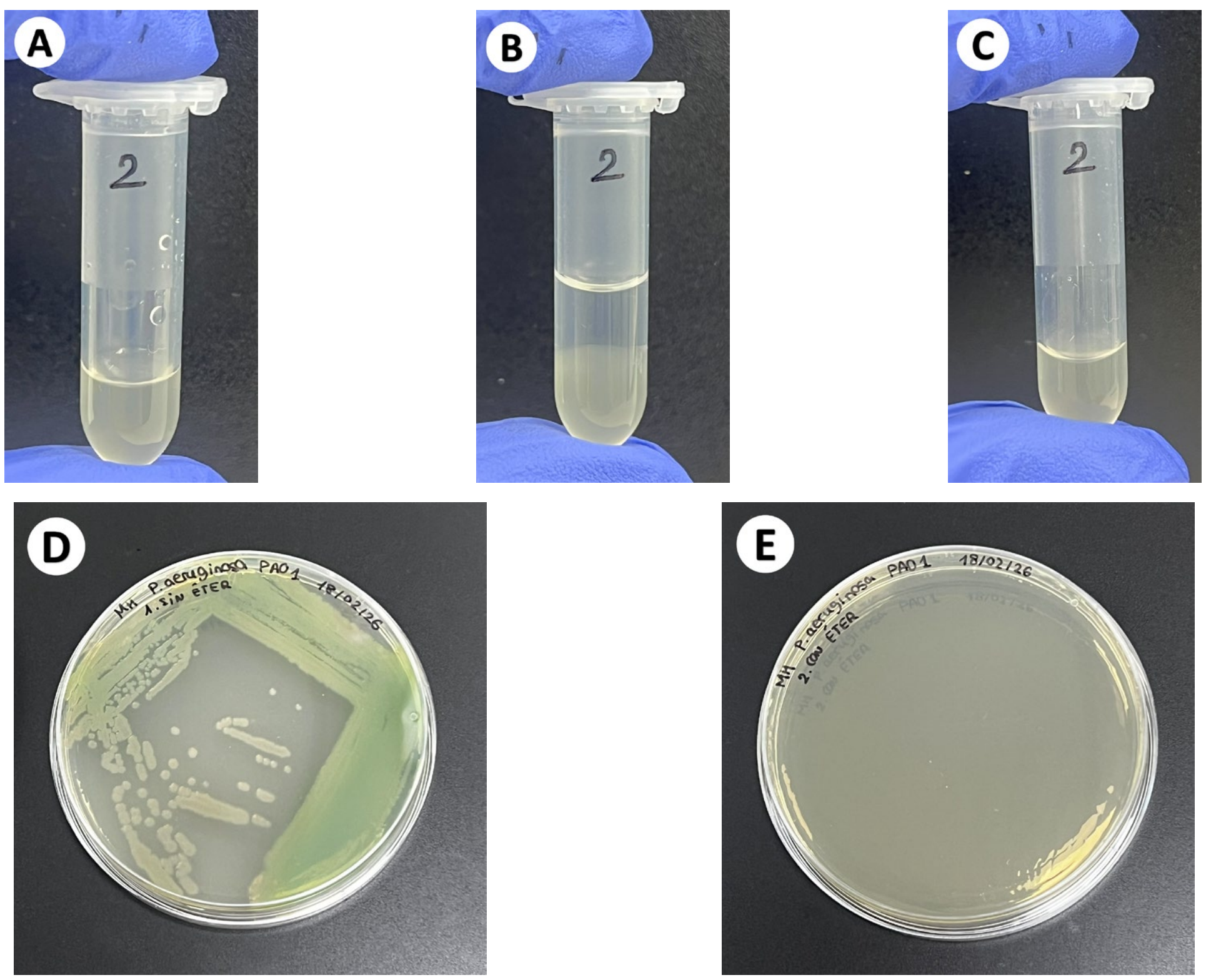

Figure S2. Photographs of the bacterial sample (A) before, (B) during, and (C) after ether treatment. Panels D and E show two cultures, with and without ether treatment, respectively, before 24 h of incubation at 37 °C.

3. EC-SERS measurements.

As described in Spectroelectrochemistry (SEC) measurements subsection, the electrochemical methodology was divided into two consecutive stages.

First, the working electrode (WE) was preconditioned in an electrolyte containing 0.1 M $LiClO_4$ and 10 mM KCl. This pre-treatment was used to activate and roughen the electrode surface and facilitate the formation of the SERS-active nanostructures. The electrode was then rinsed with the same electrolyte media, dried, and used for measurements in the same supporting electrolyte (0.1 M $LiClO_4$ + 10 mM KCl) after adding the corresponding amount of pyocyanin (PYO).

Depending on the type of information to be extracted from the experiment, two different methodologies were considered. Both differ exclusively in the choice of measurement technique, namely linear sweep voltammetry (LSV) or cyclic voltammetry (CV), thereby defining two protocols that are graphically represented in Figure S3A and Figure S3B.

As shown in Figure S3, both methodologies were divided into individual segments labelled with letters to enable an unambiguous description of the full protocol:

- **Pre-treatment step.** The first step comprised two procedures (A and B), which were carried out in 0.1 M $LiClO_4$ and 10 mM KCl.
- **Measurement step.** This second step comprised two different procedures (D or E), which were carried out in 0.1 M $LiClO_4$, 10 mM KCl, and different PYO concentrations.
  - **A. Equilibrium time.** A 5 s equilibration time at +0.20 V was applied before starting the experiment.
  - **B. Pre-conditioning cyclic voltammetry.** Cyclic voltammetry (CV) was performed between the vertex potentials of +0.20 V and -0.20 V, starting at +0.20 V in the cathodic direction with a scan rate of 0.02 $V \cdot s^{-1}$, applying two consecutive cycles.
  - **C. Cleaning process.** Between the pre-treatment and measurement steps, the electrode surface was rinsed with deionized water**.**
  - **D. Linear sweep voltammetry measurements.** Linear sweep voltammetry (LSV) was performed between the vertex potentials of +0.20 V and -0.50 V, starting at +0.20 V in the cathodic direction with a scan rate of 0.02 $V \cdot s^{-1}$.

- **E: Cyclic voltammetry measurements.** CV was performed between the vertex potentials of +0.20 V and -0.50 V, starting at +0.20 V in the cathodic direction with a scan rate of 0.02 $V \cdot s^{-1}$.

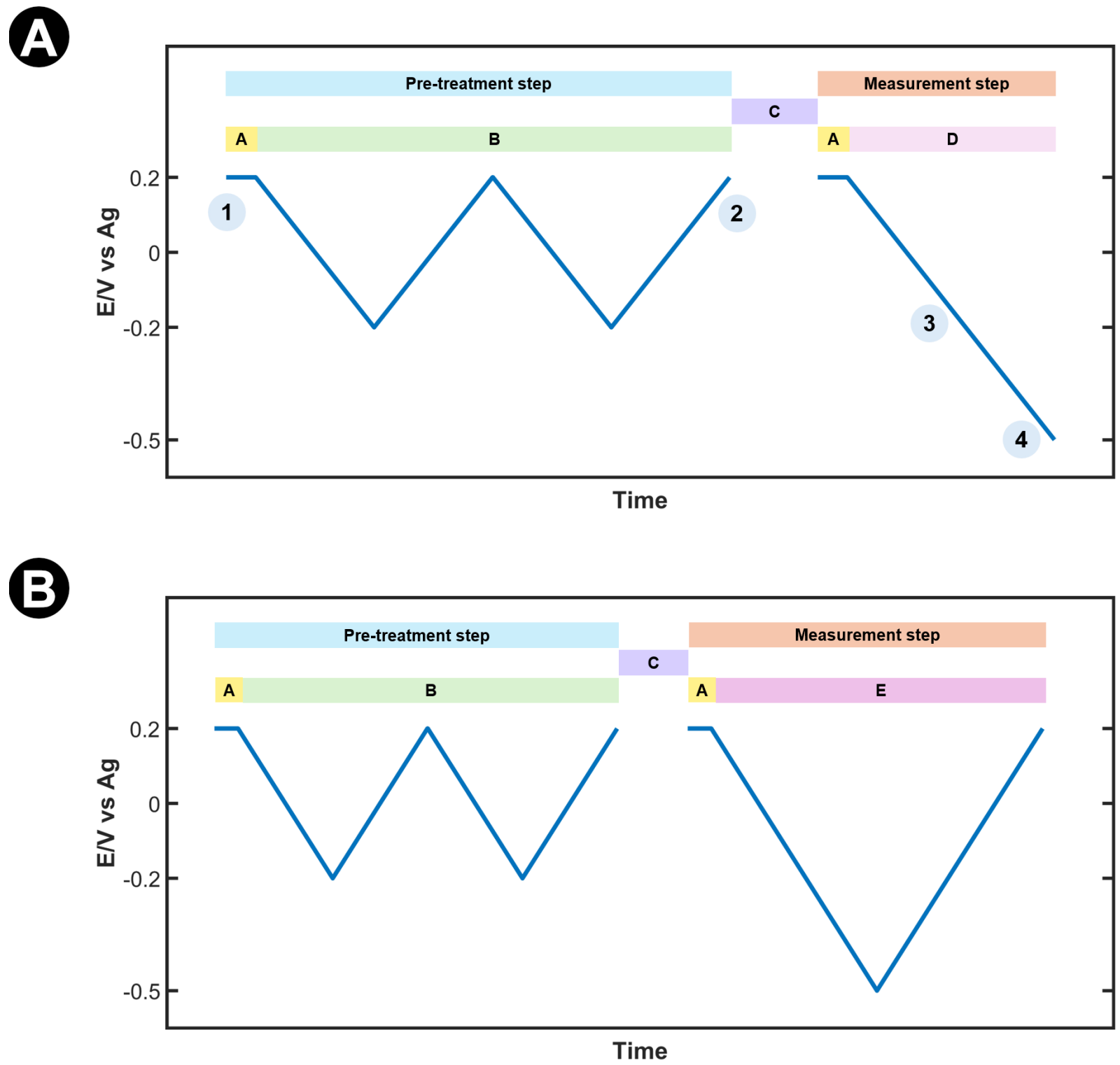

Figure S3. Graphical representation of the electrochemical methodology used to obtain spectroelectrochemical data, indicating the various stages of the pre-treatment and measurement steps with letters and the location where the samples were taken for analysis by scanning electron microscopy (SEM) with numbers. Both figures share the same sample pre-treatment prior to the measurement step, but differ slightly in the electrochemical procedure used during the measurement. Figure S3A shows the LSV measurement, and Figure S3B shows the CV measurement. In both cases, the potential limits were the same, ranging from +0.20 V to −0.50 V.

4. Assignment of Raman bands for pyocyanin.

Table S1. Assignment of Raman bands for pyocyanin[1].

SERS Raman shift (cm^{-1})	Experimental SERS Raman shift (cm^{-1})	Assignment[1]
**409**	-	Ring deformation
**490**	-	Ring deformation
**509**	-	Ring deformation
**539**	538	Ring deformation
**593**	602	Ring deformation
**635**	644	Ring deformation
**670**	678	Ring deformation
**1171**	1170	N–CH_3 wagging
**1350**	1354	C-N stretching
**1591**	1598	C-C stretching vibration
**1614**	1612	C-C stretching vibration, ring stretching

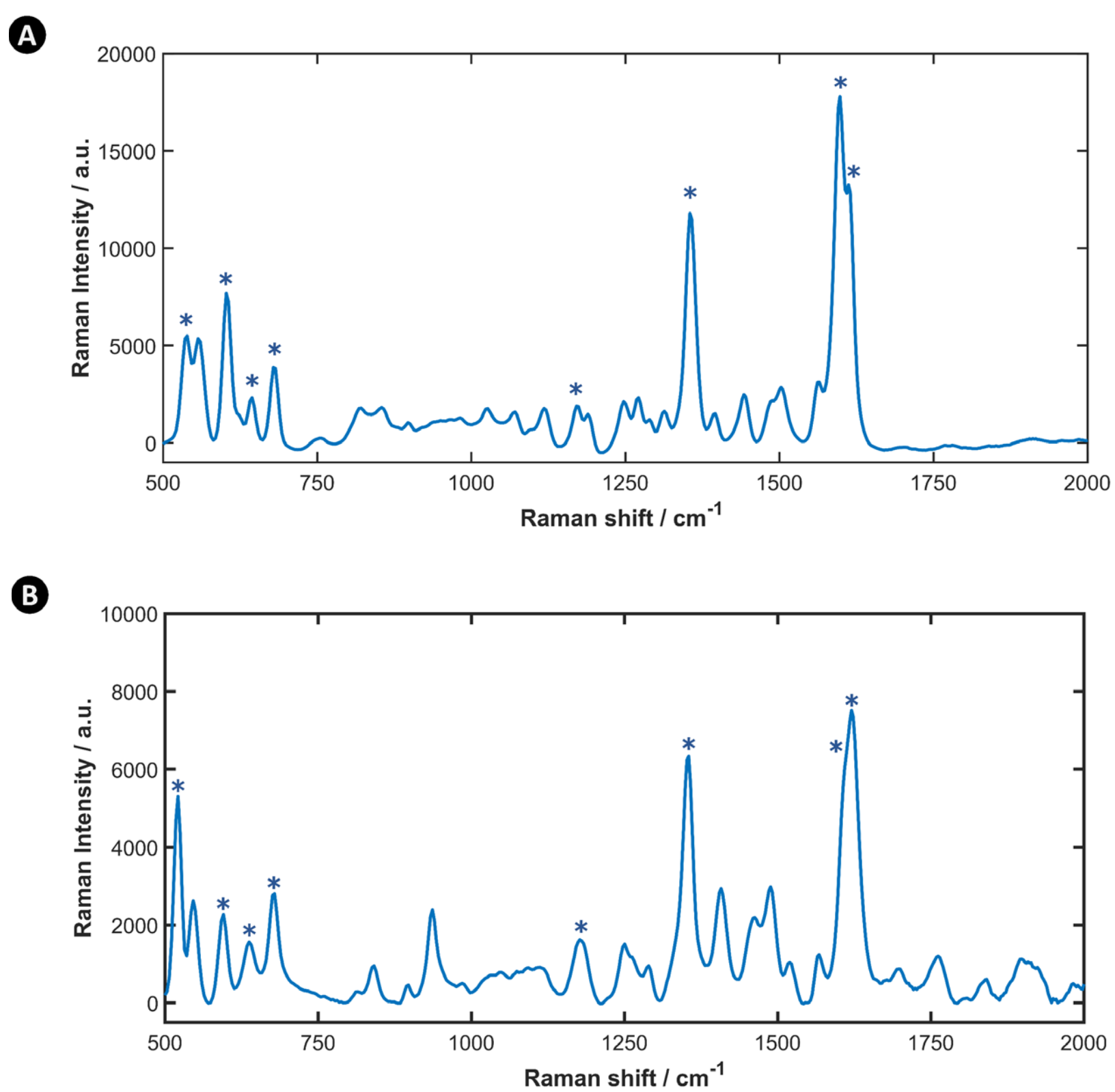

Figure S4. (A) SERS spectrum of 55.5 nM PYO recorded at −0.30 V during the cathodic scan under the experimental conditions described in the measurement protocol. The sample contained BRB (pH = 6), 0.1 M $LiClO_4$, and 10 mM KCl, and the integration time was 1 s. The characteristic Raman bands listed in Table S1 are marked in the spectrum. (B) Normal Raman spectrum of 1.44 mM PYO recorded in BRB (pH = 6) containing 0.1 M $LiClO_4$ and 10 mM KCl, with an integration time of 10 s.

5. Formal potentials of phenazine derivatives.

Table S2. Summary of experimentally determined and theoretically calculated formal potentials for phenazine derivatives. Shaded cells indicate experimentally determined values, whereas unshaded cells correspond to theoretically calculated values. To facilitate comparison, all potentials are reported versus Ag/AgCl. When only values referenced to SHE were available, they were converted from the corresponding SHE value to the Ag/AgCl scale.

Molecule	Formal potential V (vs SHE)	Formal potential V (vs Ag/AgCl)	Reference
**PYO**	-	-0.40	This work
**PZ (Phenazine)**	-0.64	-0.84	[2,3]
	-0.36	-0.56	
	-0.36	-0.56	
**DHP (2,3-dihydroxyphenazine)**	-1.19	-1.39	[2,3]
	-0.82	-1.02	
	-0.89	-1.09	
**2-HP (2-hydroxyphenazine)**	-0.57	-0.77	[3,4]
	-0.79	-0.99	
	-0.67	-0.87	
**DHPS (7,8-dihydroxyphenazine-2-sulfonic acid)**	-1.05	-1.25	[2,3]
	-0.86	-1.06	
	-0.86	-1.06	
**DHPC (7,8-dihydroxyphenazine-2-carboxylic acid)**	-1.14	-1.34	[2,3]
	-0.88	-1.08	
	-0.89	-1.09	
**PMS (Phenazine methosulfate)**	-	+0.01	[5]
	-	-0.12	
**MPMS (5-Methylphenazinium methyl sulfate)**	-	+0.01	[5]
	-	-0.11	
**5,10-Dimethylphenazinium diiodide**	-	+0.11	[5]
	-	-0.07	

6. Raman and UV/Vis spectroelectrochemical behavior of pyocyanin.

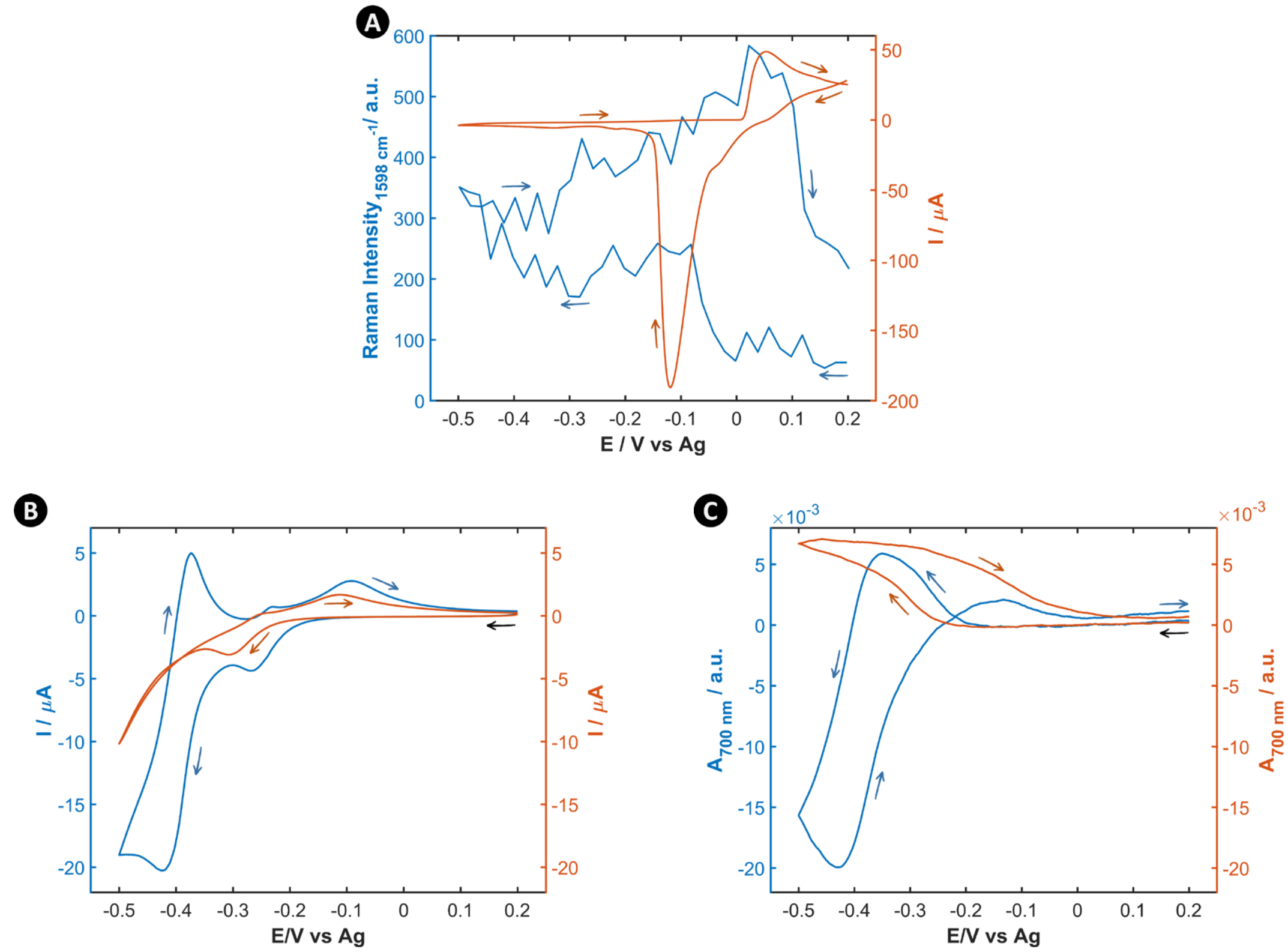

Figure S5. (A) CV (orange line) and voltaRamangram at 1598 cm^{-1} (blue line) for a sample composed by BRB (pH = 6), 0.1 M $LiClO_4$, and 10 mM KCl using an Ag-SPE. The CV started at +0.20 V in the cathodic direction down to the vertex potentials (−0.50 V) at a scan rate of 0.02 V·s^{-1}, after applying an initial potential of +0.20 V for 5 s. And comparison of (B) the CVs and (C) the voltabsorptogram at 700 nm recorded in BRB (pH = 6) and 0.1 M $LiClO_4$ media using a Au-SPE in the absence (orange line) and presence (blue line) of 555 µM PYO, respectively.

7. Surface characterization.

As shown in Figure S3, four samples were analyzed using scanning electron microscopy (SEM).

1) The first sample studied represents the surface of the commercial Ag-SPE in its initial state, without any modification (Figure S6 A-B).
2) The second sample analyzed represents the surface state of the Ag-SPE after finishing the pre-treatment step, ending at +0.20 V (Figure 2 A-B).
3) The third sample was collected during the measurement step at −0.30 V during the LSV. This point was selected to assess the onset of nanoparticle formation on the electrode surface under the applied potential (Figure 2 C-D).
4) The fourth sample was collected at the end of the LSV at −0.50 V in the measurement step. This condition was chosen to verify that the Ag NPs formed remained stable and did not undergo significant morphological changes, which is critical for the consistent Raman enhancement of the target molecule (Figure S6 C-D).

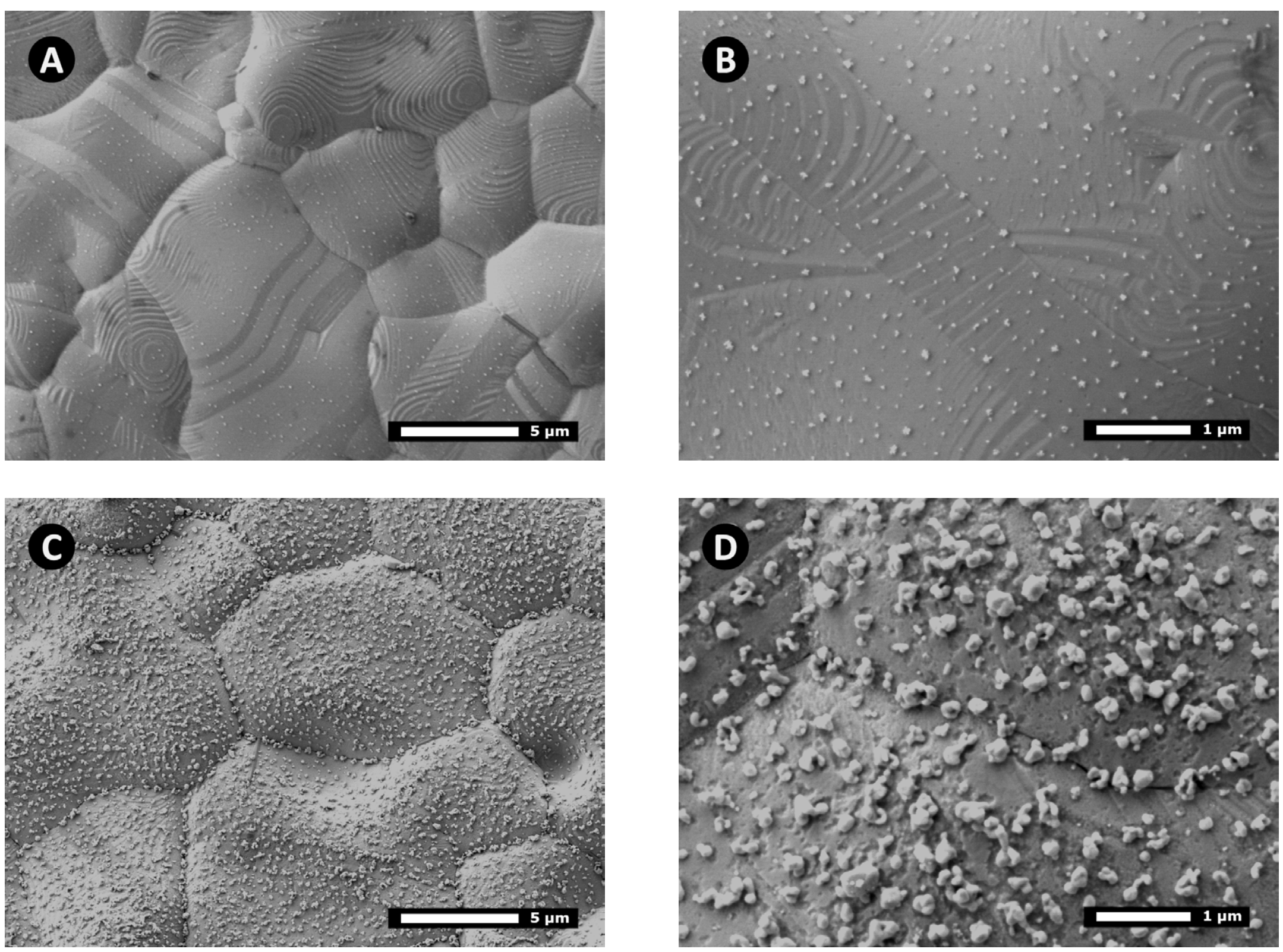

Figure S6. SEM images of the Ag-SPE surface during the pre-treatment step in a SEC experiment. (A, B) SEM images of the Ag-SPE surface at different scales without any modifications. (C, D) SEM images at different scales of the Ag-SPE surface taken at -0.50 V, once the pre-treatment step was completed.

8. Particle size distribution.

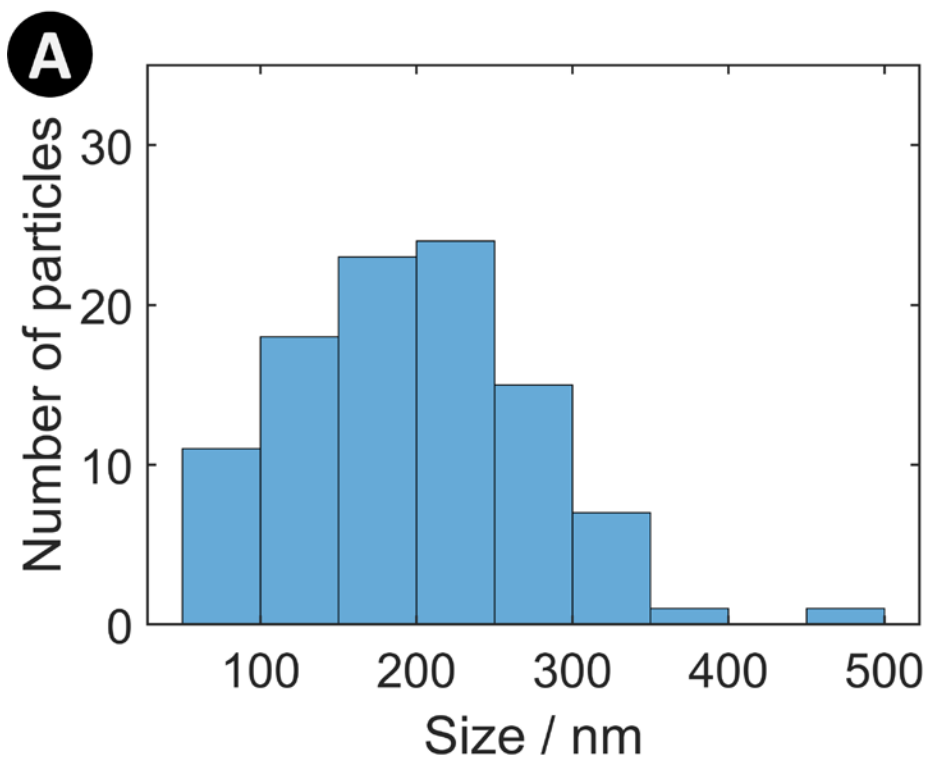

Figure S7. Particle size distribution histograms obtained from SEM images. (A) Histogram corresponding to AgCl NCs. (B) Histogram corresponding to Ag NPs.

9. pH Study.

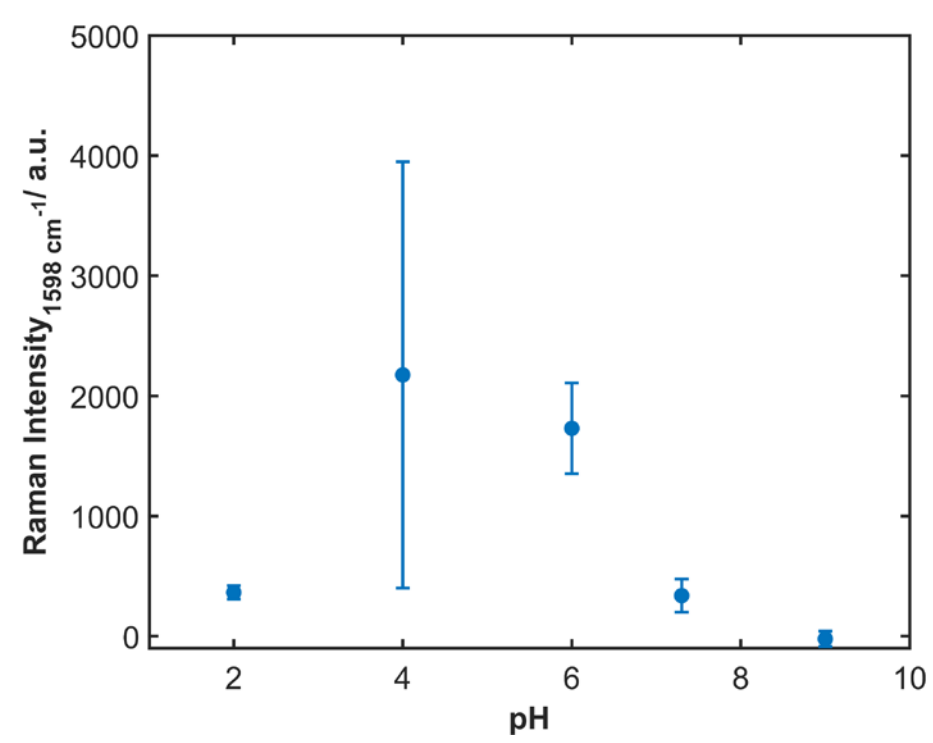

Figure S8. Effect of pH on EC-SERRS performance for PYO detection. The blue traces show the average Raman intensity of the 1598 cm^{-1} band measured at each pH value. Each measurement was performed three times, except for the samples at pH 4 and 6, which were measured five times. In all cases, the measurements were performed using a solution containing 2 nM PYO, 10 mM KCl, 0.1 M $HClO_4$, and BRB adjusted to the corresponding pH.

10. SERRS Integration time for the calibration curve.

Table S3. SERRS Integration time for each PYO sample**.**

[PYO] (nM)	$t_{integration}$ (ms)
**0.37**	4000
**0.74**	2000
**0.98**	2000
**5.55**	1000
**18.50**	500
**29.60**	200
**55.50**	200
**101.75**	100

11. Interference study (I).

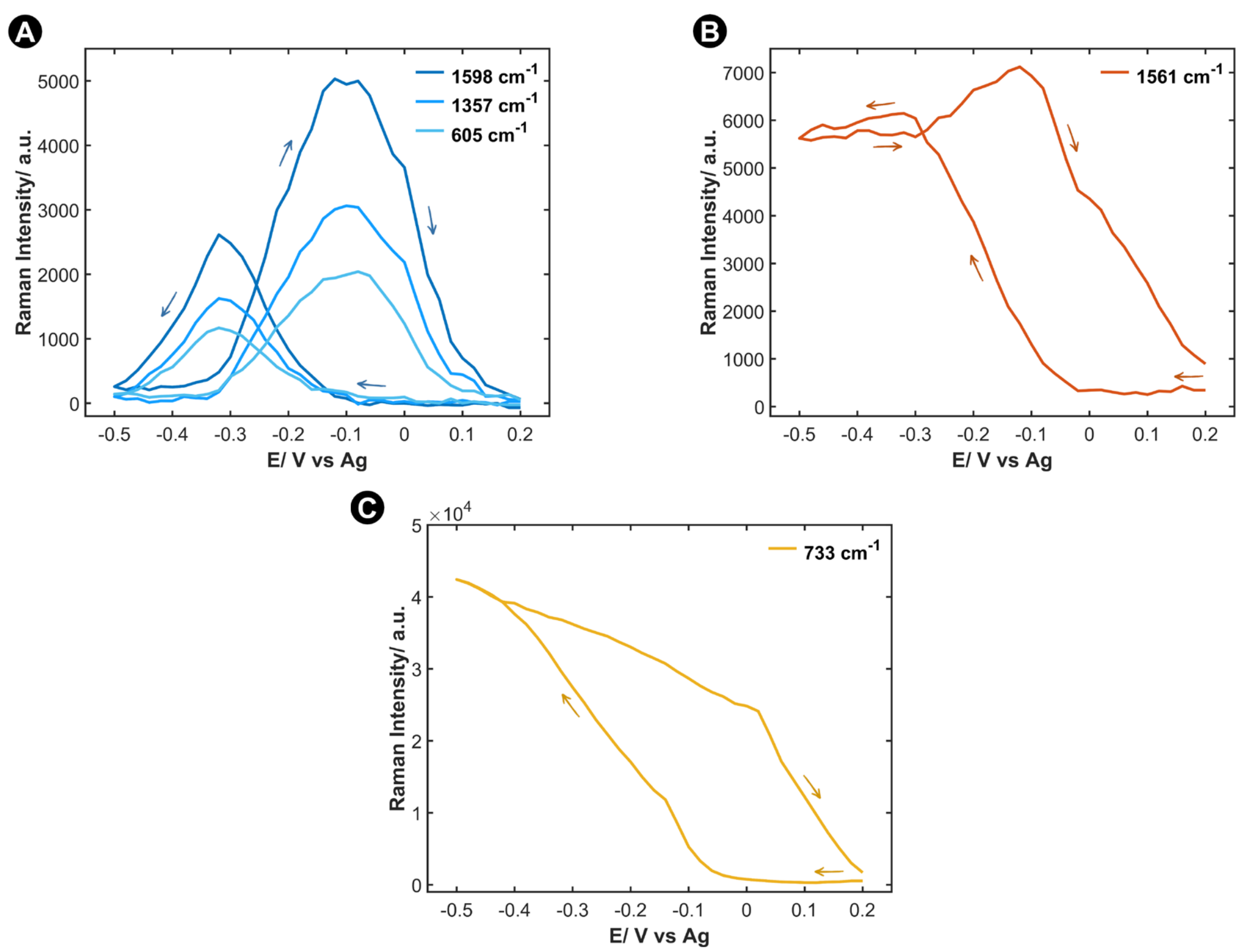

Figure S9. VoltaRamangrams of PYO were recorded at 1598, 1357, and 605 cm^{-1}. (B) VoltaRamangram of folic acid recorded at 1561 cm^{-1}. (C) VoltaRamangram of adenine recorded at 733 cm^{-1}. All samples were prepared in a medium containing 10 mM KCl, 0.1 M $LiClO_4$, and BRB (pH = 6). The electrochemical protocol used for all measurements is described in Figure S2B. The analyte concentrations were 5 nM for PYO and 5 µM for folic acid or adenine.

12. Interference study (II).

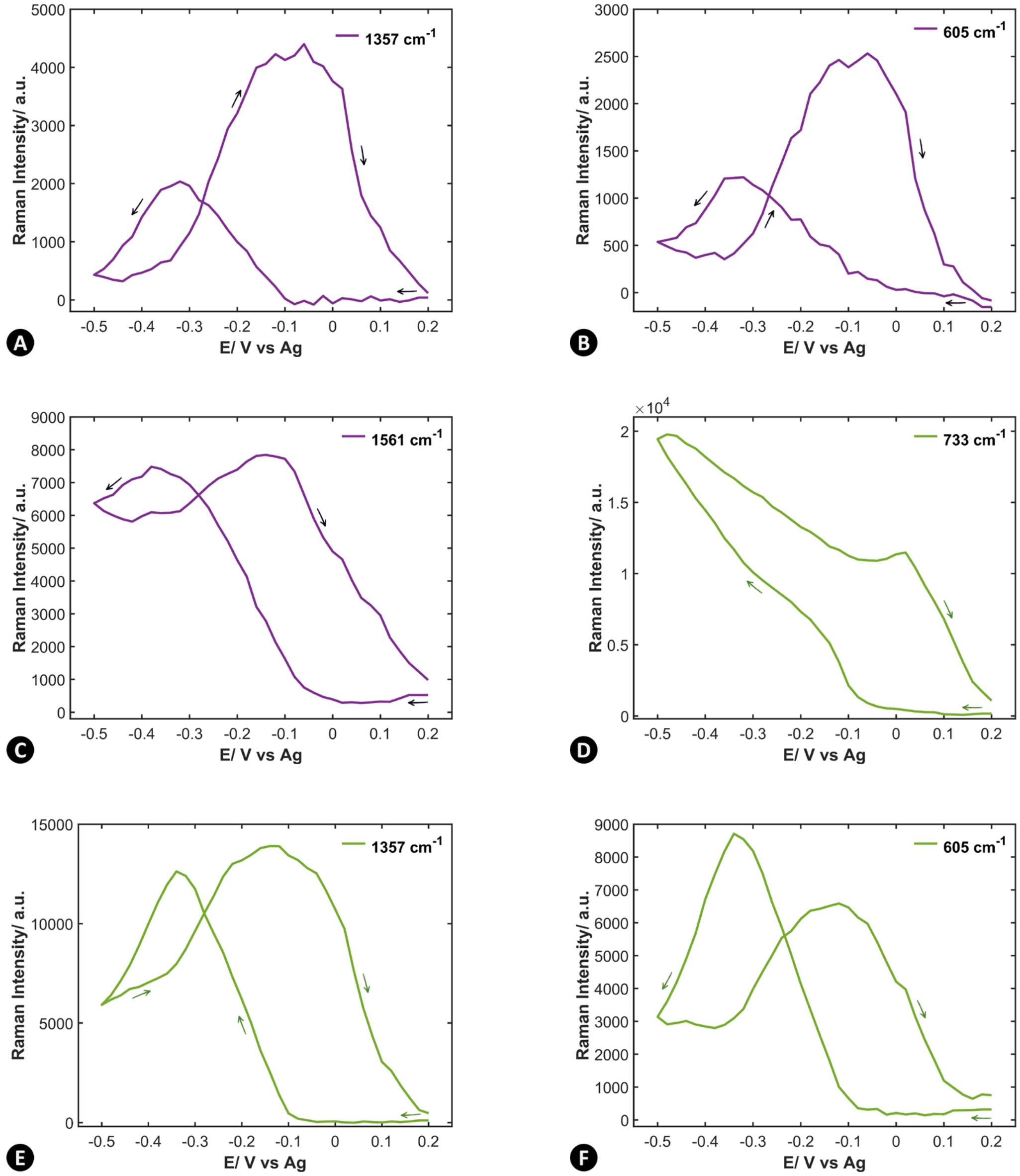

Figure S10. VoltaRamangram at 1561 cm^{-1} corresponding to folic acid in the folic acid/PYO mixture (A), and voltaRamangrams at 1357 cm^{-1} (B) and 605 cm^{-1} (C) corresponding to PYO in the same mixture. VoltaRamangram at 733 cm^{-1} corresponding to adenine in the adenine/PYO mixture (D), and voltaRamangrams at 1357 cm^{-1} (E) and 605 cm^{-1} (F) corresponding to PYO in the same mixture. All mixtures were prepared in a medium containing 10 mM KCl, 0.1 M $LiClO_4$, and BRB (pH = 6), and the electrochemical protocol used for all measurements is described in Figure S2B. The analyte concentrations were 5 nM PYO and 5 µM folic acid for the folic acid/PYO mixture and 5 nM PYO and 5 µM adenine for the adenine/PYO mixture.

13. Linear regression curves for complex matrices.

Table S4. Figures of merit for the regression obtained for each matrix**.**

Sample	Slope (nM)	Intercept (a.u.)	S_{yx}	R^2	LOD (nM)
**Tap water**	$3.665 \cdot 10^4$	$-0.508 \cdot 10^4$	$1.979 \cdot 10^4$	0.984	1.78
**Urine**	$2.945 \cdot 10^4$	$1.478 \cdot 10^4$	$1.197 \cdot 10^4$	0.993	1.34
**Saliva**	$1.201 \cdot 10^4$	$-0.426 \cdot 10^4$	$8.256 \cdot 10^3$	0.982	2.27
**Serum**	$8.935 \cdot 10^3$	$-1.139 \cdot 10^3$	$2.564 \cdot 10^3$	0.997	0.95

The LOD in Table S4 were calculated for the diluted samples. The LOD in the test sample before dilution is indicated in the main text.

14. Pyocyanin secretion.

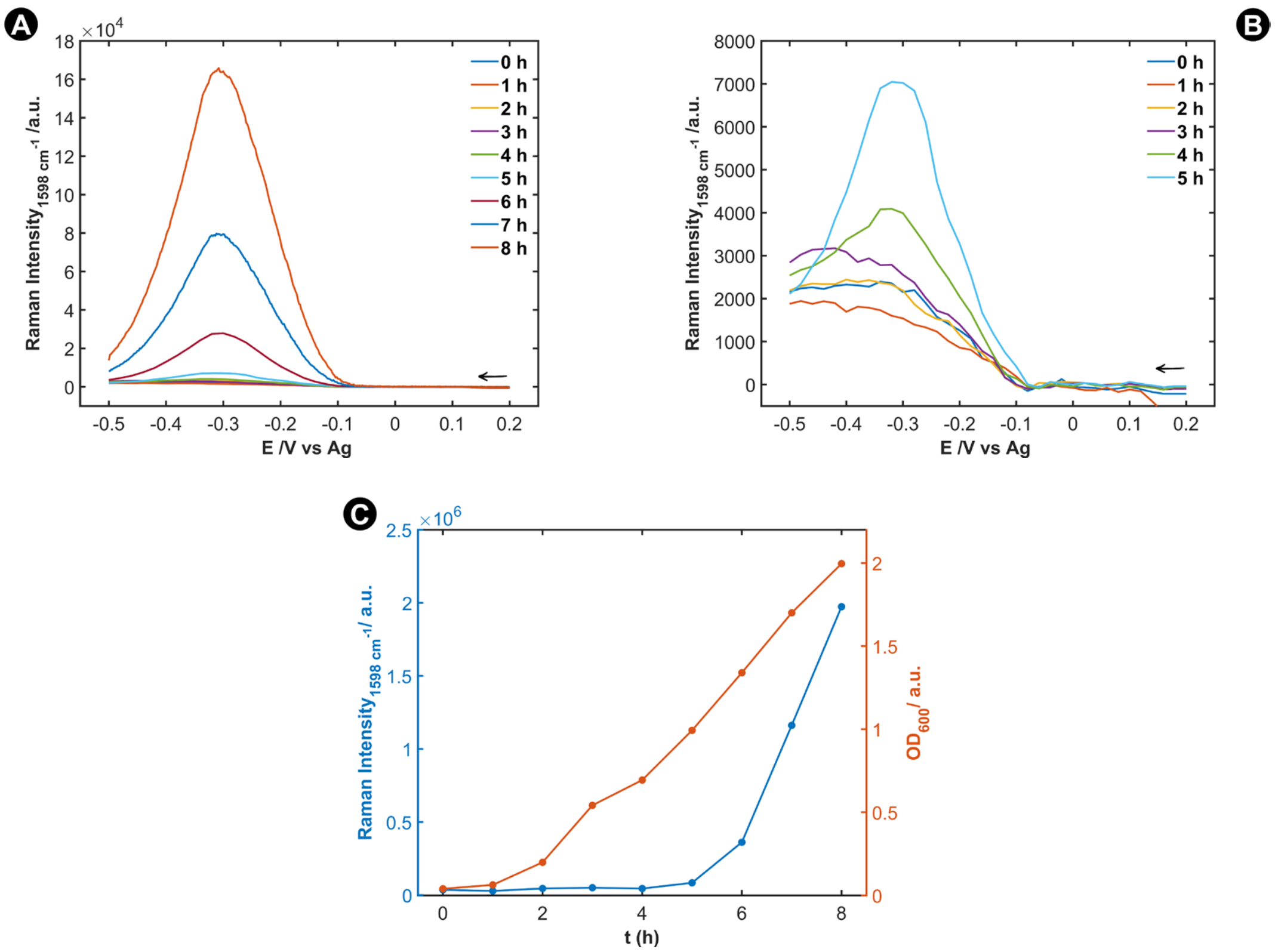

Figure S11. (A) VoltaRamangrams at different culture times (0–8 h) for aliquots collected from the microbial culture. (B) Magnified view of the behavior observed during the first 5 h. (C) Evolution of the Raman intensity of the PYO band at 1598 cm^{-1} together with the evolution of the OD_{600} measured before the sample analysis.

15. References.

(1) Liu, L.; Ma, W.; Tan, H.; Ge, C.; Zhang, B.; Wang, C.; Zhang, Y.; Xu, Y. In Situ Monitoring of Quorum Sensing Signalling Molecules Using a SERS Chip with a Micro -Chamber Array. *Anal.* **2024**, *150* (2), 318–328. https://doi.org/10.1039/d4an00992d.

(2) Hollas, A.; Wei, X.; Murugesan, V.; Nie, Z.; Li, B.; Reed, D.; Liu, J.; Sprenkle, V.; Wang, W. A Biomimetic High-Capacity Phenazine-Based Anolyte for Aqueous Organic Redox Flow Batteries. *Nat. Energy* **2018**, *3* (6), 508–514. https://doi.org/10.1038/s41560-018-0167-3.

(3) de la Cruz, C.; Sanz, R.; Suárez, A.; Ventosa, E.; Marcilla, R.; Mavrandonakis, A. A Systematic Study on the Redox Potentials of Phenazine-Derivatives in Aqueous Media: A Combined Computational and Experimental Work. *ChemSusChem* **2023**, *16* (8). https://doi.org/10.1002/cssc.202201984.

(4) Wang, C.; Li, X.; Yu, B.; Wang, Y.; Yang, Z.; Wang, H.; Lin, H.; Ma, J.; Li, G.; Jin, Z. Molecular Design of Fused-Ring Phenazine Derivatives for Long-Cycling Alkaline Redox Flow Batteries. *ACS Energy Lett.* **2020**, *5* (2), 411–417. https://doi.org/10.1021/acsenergylett.9b02676.

(5) Liu, J.; Yang, T.; Li, X.; Zhang, Y.; Dong, K.; Huang, Q.; Gan, Y.; Fan, W.; Qin, Y.; Liu, Y.; Gao, J. An Electrochemical 3-Hydroxybutyrate Biosensor Using Phenazine-Based Electron Mediators. *Talanta* **2026**, *302*, 129408. https://doi.org/10.1016/j.talanta.2026.129408.
